# Computational modelling of cell identity

**DOI:** 10.1042/BCJ20250132

**Published:** 2026-05-22

**Authors:** Woo Jun Shim, Chris Siu Yeung Chow, Shaine Chenxin Bao, Qiongyi Zhao, Nathan J. Palpant

**Affiliations:** Institute for Molecular Bioscience, The University of Queensland, Brisbane, Australia

**Keywords:** bioinformatics, cell identity, computational models, gene expression and regulation, machine learning

## Abstract

Deciphering cell identity remains a central challenge in biology, as experimental profiling can only capture a fraction of the molecular diversity across human cell states. Computational modelling fills this gap by offering scalable predictions beyond what experimental assays can measure. These models play key roles in classifying cell type, discovering previously unknown states, interpreting perturbation responses, and generating hypotheses for contexts that are experimentally inaccessible. The rapid expansion of consortium-level data has enabled models to learn generalisable genomic features of cell identity. In the present review, we examine 43 representative computational methods utilising information extracted from genomic regulatory elements, marker genes, or reference atlases and traditional machine learning or transformer-based approaches for cell identity inference. By outlining biological rationales, applications, and limitations of these methods in a minimally technical tone, the review serves as a practical guide for biologists to choose appropriate methods for their specific analytical needs.

## Introduction

Mechanisms governing cell identity are fundamental to every aspect of biological research. However, deciphering how a single shared genome is precisely orchestrated to generate 37 trillion cells with distinct identities in the human body remains a significant challenge. To address this, computational models can offer a tractable framework by approximating biological reality. In the context of cell identity inference, these models aim to capture the regulatory networks [1,2], gene expression patterns [3,4], and epigenomic signatures [5,6] that distinguish one cell state from another. Therefore, model performance depends on how effectively a given computational model captures the essence of cellular identity and the molecular mechanisms that govern cell fate specification.

The necessity for computational modelling in cell identity inference arises from the impracticality of assaying all possible molecular markers across all conceivable cell states. Even with advanced high-throughput technologies, limitations in the number of samples [7,8], timepoints [9], and molecular features [10] that can be simultaneously profiled create substantial gaps in our knowledge of cellular diversity. Furthermore, cellular fluidity during development [11], differentiation [12], and disease progression [13,14] means that current sequencing technologies, which provide static snapshots, cannot capture the full spectrum of possible cellular identities. In contrast, computational models can provide feasible predictions across the whole-cell identity spectrum.

But computational modelling of cell identity is not merely about predicting cellular identity. In many cases, computational models also serve a critical role in discovering unknown cell types [15,16], predicting cellular responses to perturbations [17,18], and uncovering regulatory relationships [1,2,6] that are not apparent in experimental data. Additionally, predictive models enable hypothesis generation about cellular behaviour under conditions that are experimentally challenging to test directly, such as early human development [19] or rare disease states [20].

Computational modelling of cell identity has been driven by the emergence of consortium-level data covering diverse biological contexts. Benefits of using the consortium-level data for cell identity modelling include enhanced statistical power through large sample sizes, standardised experimental protocols, and comprehensive molecular profiling that encompasses transcriptomes and epigenomes from various cell types. The scale and diversity of the consortium-level data enable models to learn generalisable patterns across experimental conditions from different biological contexts.

For these reasons, researchers have actively adopted the consortium-level data to build computational models of cell identity. While model complexity and prediction accuracy have evolved with the increasing scale of data and computational power, the overall goal of computational modelling remains unchanged: to capture genomic features that define cell identity and generalise these features to new or unseen data for accurate prediction. Recently, the surge of available tools including machine learning (ML)-based approaches has substantially broadened the landscape of computational modelling for cell identity.

The present review outlines widely adopted computational tools for modelling of cell identity. While many computational methods exist for cell identity modelling, we selected those with broad community adoption and suitability to represent four key algorithmic groups: 1. regulatory information-based, 2. marker or reference-based, 3. supervised or semi-supervised ML, and 4. transformer-based methods. Our goal is to provide succinct descriptions of the difference between these groups in terms of their biological rationale and potential caveats. We conclude the review with future directions and developments expected in this rapidly evolving field.

## Consortium-level genomic data for modelling cell identity

Data resources capturing the molecular basis of biological diversity are essential for building generalisable computational models. Consortium-level datasets require coordinated efforts to profile molecular features across diverse biological contexts from various cell lineages, tissues, and diseases. Such datasets commonly include multi-omics measurements including transcriptomics, epigenomics, and chromatin accessibility across a multitude of samples with enhanced statistical power

Furthermore, consortium-level data offer enhanced statistical power, standardised experimental protocols, uniform computational processing pipelines, and comprehensive metadata annotations that ensure data quality and reproducible computational modelling. These benefits are exemplified by the discovery of thousands of tissue-specific eQTLs and fine-mapping of causal variants from GTEx data [21] and EpiMap, an integration of over 3000 datasets from multiple consortia including Roadmap and ENCODE [22–24].

Historically, the scale of consortium-level data has evolved with advancements of sequencing technologies (Table 1). Early efforts established foundational genomic references, with the 1000 Genomes Project mapping population-scale genetic variation [25] and ENCODE annotating functional elements in the human genome [26], expanding to more than 23,000 functional genomic experiments in the latest update [24,27]. The advancement of high-throughput sequencing techniques has also accelerated the release of epigenomic databases such as the Roadmap Epigenomics project characterising 111 reference human epigenomes [23] and the International Human Epigenome Consortium [28]. Additionally, initiatives for specific biological focus also emerged, including BLUEPRINT (hematopoietic focus [29]), the Cancer Cell Line Encyclopedia profiling gene expression and variant information from 947 cancer cell lines [30], CancerSEA providing cancer single-cell state annotations [133], CistromeDB integrating large-scale TF ChIP-seq and chromatin accessibility datasets [132], and transcriptomic profiling through FANTOM’s CAGE-based transcriptional network analysis [31]. This was followed by the emergence of single-cell sequencing that has fundamentally transformed cell identity inference. These data resources include the Human Cell Atlas framework [4], Single Cell Atlas [32], brain-specific PsychENCODE [33], and multi-organ datasets such as Tabula Sapiens [34] and Tabula Muris [35]. Additionally, single-cell datasets derived from specific biological contexts can provide more focused and in-depth resources for targeted investigations, as exemplified by studies of fetal development [36,37], ageing [38,39], immune dynamics across life stages [40], and cross-species retinal comparison [41].

**Table 1:** Consortium-level genomic datasets for cell identity modelling

Consortium data (and reference)	Data types	Scale	Key contributions	Year
*BLUEPRINT* [29] https://blueprint-data.bsc.es/#!/	Transcriptomes, epigenomes	>100 blood cell epigenomes	Hematopoietic differentiation, blood disease mechanisms	2012
*The Cancer Cell Line Encyclopedia* [30] https://sites.broadinstitute.org/ccle	Transcriptomes, GWAS	947 cancer cell lines	Cancer cell-focused data resource	2012
*ENCODE* [26] https://www.encodeproject.org	Transcriptomes, epigenomes, TF ChIP-seq	More than 23,000 functional genomic experiments	Functional genome annotation, regulatory element maps	2012 (first)
*FANTOM* [31] https://fantom.gsc.riken.jp	CAGE-seq	∼3000 samples, 200,000 promoters	Transcriptional regulatory networks, enhancer dynamics	2014 (first)
*1000 Genomes* [25] https://www.internationalgenome.org	Whole-genome sequencing	2504 individuals	Foundational human genetic variation reference	2015
*Roadmap* [23] https://egg2.wustl.edu/roadmap/web_portal	Epigenomes	111 reference epigenomes	Tissue-specific chromatin states, regulatory landscapes	2015
*The International Human Epigenome Consortium* [28] https://epigenomesportal.ca/ihec	Epigenomes	7000 reference epigenomes	Reference genomes in both healthy and diseased	2016
*CistromeDB* [132] http://cistrome.org/db/#/	Epigenomes, TF ChIP-seq	13,366 human samples	Standardised curation and QC between samples	2016
*Human Cell Atlas* [4] https://www.humancellatlas.org	Transcriptomes, epigenomes	Global effort, >400 cell types	Comprehensive human cell census, cell type definitions	2017
*PsychENCODE* [33] https://www.psychencode.org	Transcriptomes, epigenomes	1866 individuals, 79,000 enhancers	Brain cell identity, psychiatric disorder mechanisms	2018
*HuBMAP* [42] https://portal.consortium.org	Single-cell multi-omics	>30 organs, 2300 datasets	Reference spatial maps of functional tissues	2019
*CancerSEA* [133] http://biocc.hrbmu.edu.cn/CancerSEA	Single-cell transcriptome, Gene signatures of 14 cancer states	41,900 cancer single cells, 25 cancer types	Extensive cancer-focused transcriptomes	2019
*GTEx* [21] https://www.gtexportal.org/home	Transcriptomes, whole-genome sequencing, eQTLs	49 tissues, 838 donors	Genetic regulation of gene expression, tissue specificity	2020
*Tabula Muris* https://tabula-muris-senis.sf.csbiohub.org	Single-cell transcriptomes	104,000 cells from 23 organs	Reference data resource for mouse cell types	2020
*Descartes* [36,37] https://descartes.brotmanbaty.org	Multi-omics single-cell	RNA-seq (4M cells from 121 tissues) ATAC-seq (720K cells from 53 tissues)	Data resource for foetal development	2020
*EpiMap* [22] https://compbio.mit.edu/epimap	Epigenomes	10,000 maps, 833 samples	Comprehensive regulatory annotation, disease variant interpretation	2021
*Tabula Sapiens* [34] https://cellxgene.csiscience.com/collections/e5f58829-1a66-40b5-a624-9046778e74f5	Single-cell transcriptomes	500,000 cells, 24 tissues	Multi-organ cell atlas, cell type characterisation	2022
Single Cell Atlas [32] https://www.singlecellatlas.org	Multi-omics single-cell	125 tissues, millions of cells	Cellular diversity, developmental programs	2024
*PanSci.* [39] https://cells.ucsc.edu/?assay=snRNA-seq&ds=mouse-pansci	Single-cell transcriptomes	20M mouse cells in 623 tissues	Ageing process, specifically in immunodeficiency context	2025
*Human Immune Health Atlas* [40] https://apps.allenimmunology.org/aifi/resources/imm-health-atlas/	Single-cell transcriptomes	1.8M cells from 108 healthy individuals	Covers multiple age groups	2025
*Mouse aging ATAC atlas* [38] https://mouseagingatacatlas.org	Single-cell ATAC-seq	>10M mouse nuclei across 21 tissue types	Chromatin accessibility data during ageing process	2026

In parallel with the development of sequencing techniques, advances in computational integration have enabled efficient cross-dataset analysis and streamlined data processing across multiple databases. For example, the EpiMap database imputes data across 10,000 epigenomic maps [22]; GTEx integrates RNA sequencing and genotypes to calculate eQTLs [21]; and more recently, HuBMAP has leveraged the development of spatial and single-cell multi-omics to study tissue organisation [42].

Consortium-level data provide a critical knowledge base for building computational models capable of extracting representative cell identity patterns. However, several limitations of consortium-level data need to be considered in terms of leveraging their utility for modelling purposes. Firstly, many consortia-level data resources cover only a limited number of data modalities (e.g. RNA versus epigenomic versus DNA), which restricts the use in forming a holistic view of cell identity. Secondly, the scope of biological diversity is limited, resulting in ascertainment biases that inherently affect model generalisability. The present issue is particularly important when contextualising the model’s predictions in rare cell or disease conditions that are not sufficiently covered by much consortium-level data.

## Computational tools for studying cell identity

Computational methods for studying cell identity inference generally fall into one of four groups based on their design: 1. regulatory information-based methods, which infer cell identity from underlying transcriptional control logic (e.g. epigenetics, transcription factors (TFs), and gene regulatory networks (GRNs)), 2. marker or reference-based methods, which rely on pre-defined cell-type signatures or annotated atlases without requiring ML approaches, 3. supervised or semi-supervised ML-based methods, which require ML training on reference data with fully or partially known ground truth to learn decision boundaries or latent representations of cell identity, and 4. transformer-based methods, which leverage self-supervised pre-training with attention mechanisms to capture complex dependencies between biological units.

To assess each method’s utility, we first defined five key tasks closely associated with cell-type modelling; 1. cell-type annotation (i.e. can the method directly assign cell types?), 2. regulatory inference (i.e. can the method identify key regulatory genes or GRNs?), 3. cell fate dynamics (i.e. can the method infer cell reprogramming, trajectories, or rare cell states?), 4. data integration (i.e. can the method harmonise and integrate multiple datasets?), and 5. perturbation modelling (i.e. can the method simulate perturbation experiments, including drug response or TF perturbations?). These tasks were defined to reflect each method’s primary functional capacity, rather than secondary or indirect uses (e.g. while some tools may support downstream analyses through exported outputs, they are not considered to directly perform those tasks). Second, we assessed each method’s usability by three criteria: 1. source code availability (i.e. whether a repository for source code exists), 2. tutorial documentation (i.e. whether detailed implementation guidance is provided), and 3. cloud accessibility (i.e. whether an interactive web-based interface is available) (full links are provided in Supplementary Table S1). Lastly, we surveyed each method’s implementation convenience by examining 1. the programming languages used (R or Python) and 2. whether a single command-line execution is supported. Each method was manually reviewed against these criteria, and the results are summarised in a table to help users to guide method selection based on their analytical needs and computational expertise. While not exhaustive, these criteria capture the core functionalities of computational methods for cell identity.

To further assist with implementation and method selection, we summarised the required input data, expected outputs, and unique features of each tool in a separate table. To assist with interpreting ML terms for non-experts, we provide a glossary of terms in Table 2.

**Table 2:** Glossary

Term	Description
**Attention mechanism**	A feature that lets a model focus on the most important parts of the input data when making a prediction.
**Autoencoder**	A type of neural network that learns to compress data into a smaller representation and then reconstruct the original data from that compression.
**BERT (bidirectional encoder representations from transformers)**	A model that reads sentences and other text in both directions to extract meaning of context.
**Contrastive learning**	A training method where the model learns by contrasting positive examples (similar data points) with negative examples (dissimilar data points).
**Decoder**	A part of autoencoder that takes a compressed representation and converts it back into an interpretable output.
**Embedding**	A numerical representation of a piece of data (like a word or a cell profile) that captures its meaning and relationships with other pieces of data.
**Encoder**	A part of autoencoder that takes the original data and compresses it into a smaller, meaningful representation.
**Ensemble method**	A technique that combines the predictions of several individual machine learning models to get a more accurate overall result.
**Generative model**	A model designed to create new data that resembles the data it was trained on.
**GPT (generative pre-trained Transformer)**	A model designed to generate human-like text by predicting the next most likely word in a sequence.
**LLM (large language model)**	A massive, advanced AI model, typically based on the transformer architecture, that is trained on huge amounts of text to understand and generate human language.
**Latent space (or dimension)**	A hidden, compressed mathematical space inside a model where important characteristics of the data are stored as numbers.
**Logistic regression model**	A simple method used for predicting a category or class, often used to determine the probability of a binary (yes/no) outcome.
**Machine learning**	The practice of teaching computers to learn patterns and make decisions or predictions directly from data without being explicitly programmed.
**Manifold**	A mathematical concept that describes the low-dimensional surface where complex high-dimensional data lies.
**NLP (natural language processing)**	A field of AI focused on enabling computers to understand, interpret, and generate human language.
**NMF (natural language processing)**	A technique used to simplify complex data by breaking it down into two smaller matrices, often representing parts and their weights.
**Nearest neighbours**	A simple technique that classifies a new data point based on the majority class of the data points closest to it.
**Neural network**	A system of interconnected processing units, inspired by the human brain, that learns to recognise complex patterns in data.
**PCA (principal component analysis)**	A statistical method used to simplify data by finding the most important underlying axes that capture the maximum variability.
**Semi-supervised method**	A training approach that uses a large amount of unlabelled data alongside a smaller amount of labelled data to improve learning efficiency.
**Supervised method**	A training approach where the model learns from data that has already been labelled with the correct answers.

### Regulatory information-based methods

**Figure 1:**
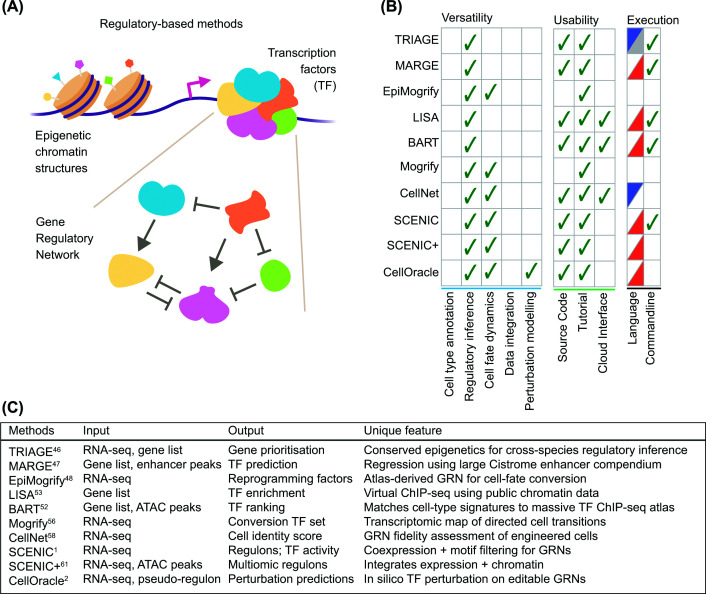
Regulatory information-based methods infer cell identity through epigenomic and gene regulatory logic. (**A**) This group utilises genomic information that regulate transcriptional outcomes, including epigenetics, transcriptional factor binding, and GRNs. (**B**) Comparison of methods for versatility, usability, and executability. Ticks indicate that a given method supports the feature. The language column denotes the programming language where the method is implemented, R (blue) or Python (red). (**C**) A summary table for input, output, and unique features of each method.

Regulatory information-based methods infer cell identity using regulatory signatures of cellular states, including chromatin features, enhancer activity, TF circuitry, or GRN (Figure 1A). These methods are based on observations that cell identity is more accurately represented by their regulatory mechanisms rather than transcriptional readouts [12,23,43–45]. Furthermore, their reliance on high-level regulatory features allows these methods to generalise across cell types and species when supported by sufficiently diverse training data. Additionally, these methods can identify mechanistic drivers of cell identity and reveal transcriptional regulators, epigenomic control elements, and cell fate determinants.

#### Epigenetic regulatory scoring methods

Methods such as TRIAGE [46], MARGE [47], and EpiMogrify [48] infer regulatory determinants of cell identity by extracting biologically meaningful epigenetic signals embedded in global chromatin states. These approaches rely on two key assumptions: regulators of cell identity exhibit characteristic epigenetic signatures across many tissue and cell types, and these signatures are detectable only when projected on large-scale data covering broad biological diversity.

TRIAGE [46] is driven by a simple but powerful rationale; developmentally regulated genes accumulate broad H3K27me3 domains across a wide range of cell types. This rationale reflects a conserved PRC2 (Polycomb Repressive Complex 2)-mediated mechanism that represses potent cell identity regulators except in cell types and states where they are required. TRIAGE quantifies each gene’s propensity to acquire broad H3K27me3 across hundreds of cell types from Roadmap, EpiMap, and ENCODE, into a single metric called the repressive tendency score (RTS). This score can be directly applied to gene expression data to prioritise regulatory genes specific to a given biological context. Furthermore, the RTS enables cross-species discovery of regulatory genes and provides a regulatory axis that is often more stable than expression itself. The RTS has shown its utility in clustering single cells and functional gene groups [49] and characterisation of genome loci at base-pair resolution [50]. However, its sole dependence on H3K27me3 means that the RTS overlooks non-PRC2 regulatory mechanisms. Therefore, cell states that deviate from canonical PRC2 behaviour may be poorly represented in this metric.

MARGE [47] quantifies a gene’s regulatory potential (RP) from proximal H3K27ac-marked regions. It models the H3K27ac-to-gene relationship using an exponential distance function and aggregates these patterns across hundreds of publicly available samples, resulting in an RP calculation that quantifies how strongly a given gene is regulated by H3K27ac. This enhancer-driven backbone [51] allows MARGE to infer cell-type-specific master regulators through the RP, serving as a foundational method for downstream approaches like BART [52] and LISA [53] (discussed below). However, its dependence on a simplistic distance-based gene linking algorithm limits its accuracy, as chromatin topology frequently violates this proximity assumption [54]. Furthermore, TF ChIP-seq data used are context-mismatched in some cases, and this can potentially mislead their biological associations.

EpiMogrify [48] models cell identity through H3K4me3 breadth. It builds on earlier findings that broad H3K4me3 correlates with transcriptional stability and regulatory potency of genes [55]. Hence, the method assumes that any changes in peak breadth across tissues reflect reconfiguration of regulatory control during cell differentiation. EpiMogrify uses these shifts to prioritise TFs that modulate cell fate transitions and provides a regulatory ranking tailored for cell reprogramming applications. Like TRIAGE and MARGE, however, its reliance on a single epigenetic feature remains a central limitation.

#### Transcription factor-centric regulatory inference methods

LISA [53] and BART [52] utilises publicly available TF ChIP-seq libraries to infer transcriptional regulation in the genome. LISA [53] first constructs a RP landscape by integrating DNase and histone modification data. Subsequently, it simulates the removal of each TF's binding profile to determine which TFs most strongly support the regulation of an input gene set. This *in silico* TF deletion strategy provides interpretable outputs; TFs whose removal destabilises the gene set are inferred as regulators. LISA is especially useful for identifying lineage regulators in contexts where differential expression alone fails to reveal upstream drivers. Its major limitations stem from reliance on sparsely profiled public TF datasets. This sparsity means that the perturbation analysis could originate from unrelated cell types, and combinatorial TF behaviour might not be adequately modelled.

In comparison, BART [52] uses a simpler and faster strategy; it computes the association between enhancer regions linked to a gene set of interest, which is derived from MARGE’s RP, and the TF binding profiles from large ChIP-seq compendia. TFs whose binding patterns most consistently overlap these enhancer regions are identified as likely regulators. The method can efficiently identify candidate TFs controlling gene expression modules, perturbation signatures, or scRNA-seq cluster-specific genes. Unfortunately, it inherits the same limitations of the H3K27ac-gene linking model from MARGE. Furthermore, it is strongly influenced by the uneven representation of TFs in public datasets (i.e. TFs with extensive ChIP-seq coverage tend to dominate rankings). Nonetheless, statistical stability of LISA and BART and their ability to recover plausible regulators are only possible because of the breadth of TF ChIP-seq data offered by ENCODE and Cistrome DB.

#### Gene regulatory network-based methods

GRN is a set of interacting molecules that control gene expression within a cell. Methods in this sub-category use the GRN as the unit of cell identity. For instance, Mogrify [56] aims to identify TFs driving trans-differentiation through assessing GRN differences between source and target cell types. The rationale is straightforward; cell fate conversion is fundamentally a network reconfiguration problem; therefore, identifying TFs that occupy network bottlenecks would enable rational transdifferentiation strategies. Its limitations mainly arise from the noisiness of GRNs constructed from bulk RNA-seq data [57]. On the other hand, CellNet [58] uses a related but more quality-control-oriented approach. Instead of predicting TFs required for cell fate transition, CellNet assesses how well engineered cells recapitulate the GRNs of true tissues. By comparing the GRNs of induced or reprogrammed cells to reference networks derived from large transcriptomic datasets, CellNet quantifies how closely a generated cell type is matched at the regulatory level. While this method reveals deficiencies in engineered cell identities, it performs better for well-characterised tissues with high-quality reference networks.

On the contrary, SCENIC [1] takes a bottom-up approach; rather than starting with TFs, using public TF databases JASPAR [59] and TRANSFAC [60], SCENIC first identifies modules of co-expressed genes, then uses motif enrichment to infer which TFs regulate each module. This creates regulons (i.e. groups of genes) whose activity is subsequently quantified in individual cells. A key advantage of using regulons compared with individual genes is that this collective view is far more robust to transcriptional noise, especially in transitional cell states. For this reason, SCENIC is particularly useful in developmental and cancer contexts where lineage bifurcations and rare cell populations frequently emerge. However, its reliance on co-expression conflates simple correlation with causal relationships between TFs and their downstream targets. To address this limitation, SCENIC+ [61] integrates chromatin accessibility and enhancer-gene linking directly into the regulon construction. This multimodal integration better captures context-specific TF-enhancer relationships and improves the accuracy of regulon assignment. Undoubtedly, SCENIC+ benefits from the growth of single-cell ATAC (assay for transposase-accessible chromatin)-seq atlases and high-quality enhancer annotations from consortium-level data for the construction of more effective models.

Finally, CellOracle [2] models how regulatory networks would behave under *in silico* TF perturbation. It constructs a hybrid GRN by combining motif-by-accessibility priors with expression-based refinement, then simulates cell transitions by TF perturbations. This allows estimation of how perturbation of specific TFs (i.e. knocking down or over-expression) would redirect differentiation trajectories or disrupt lineage commitment. Thus, CellOracle can be particularly effective in identifying candidate TFs driving lineage bifurcations, fate decisions, and cell-state transitions.

#### Conclusion

Regulatory information-based methods offer mechanistic insight governing cell identity and generalise well across different contexts. Overall, methods that utilise GRNs tend to have strong versatility (Figure 1B), as exemplified by CellOracle, which builds comprehensive GRNs to model regulatory relationships between cell states. On the other hand, LISA, BART, and CellNet provide user-friendly environments with accessible source code, tutorials, and cloud-based interactive platforms for quick analyses. TRIAGE provides both Python and R implementations [62], accommodating researchers with different programming backgrounds. For the implementation, RNA-seq remains the most common input, while TRIAGE, MARGE, LISA, or BART can also take a pre-defined gene list or genomic loci (Figure 1C). As these methods are primarily built on transcriptional regulatory information, however, their output does not directly identify a specific cell type. Instead, they commonly return a set of predicted regulators for the cell identity or a group of co-regulated genes or regulons.

It is important to note that the effectiveness of these methods is largely restricted by the features they rely on. As a result, their predictions can miss cell states governed by noncanonical regulatory processes. Simplified assumptions about genomic loci-gene connection and regulatory causality further limit prediction accuracy. Despite these limitations, their intuitive design and focus on regulatory logic make them highly complementary to other analytical approaches, particularly when the goal is to understand mechanisms of cell identity and state transitions.

### Marker or reference-based methods

**Figure 2:**
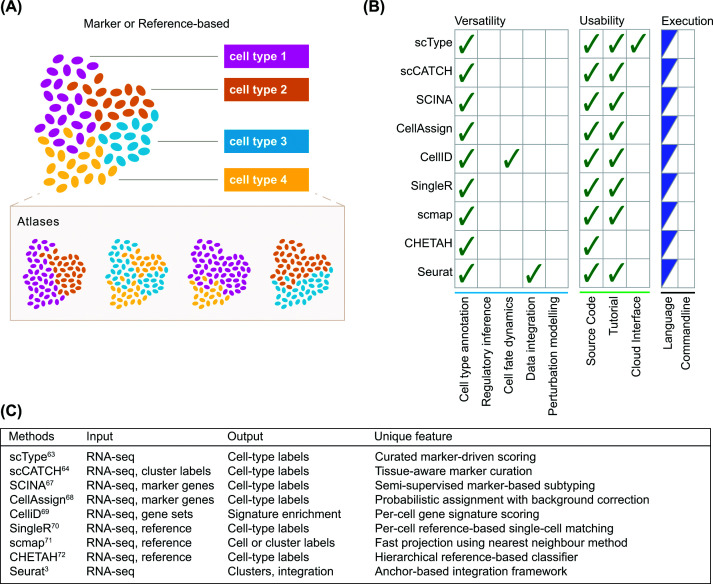
Reference-based methods assign cell identity using known gene signatures and atlases. (**A**) This group utilises knowledge from known marker genes or established atlases to perform cell type inference. (**B**) Comparison of methods for versatility, usability, and executability. Ticks indicate that a given method supports the feature. Language column denotes the programming language where the method is implemented, R (blue) or Python (red). (**C**) A summary table for input, output, and unique features of each method.

Reference-based methods represent arguably the most interpretable approaches for cell identity inference. These methods rely on established biological knowledge on curated gene sets or annotated references. This group assumes that cell identity can be identified by directly comparing a query transcriptome to known marker gene sets or well-annotated reference atlases (Figure 2A). These methods are fast and highly interpretable, but they are inherently constrained by the quality of the references.

#### Cluster-based marker enrichment models

scType [63] and scCATCH [64] embody the traditional workflow of performing unsupervised clustering of cells first based on their gene expression profiles, followed by interpretation of each cluster with curated marker gene databases. Their biological rationale is as follows: major cell types should form discrete transcriptional clusters, and these clusters should be identifiable by canonical markers of a predominant identity. Thus, both methods require cell clustering as the first step, which can introduce subjectivity in choosing cluster parameters. scType [63] collects gene sets from CellMarker [65] and PanglaoDB [66] databases. Each gene set represents a specific cell type. Then scType computes enrichment scores for these gene sets across pre-calculated cell clusters. These scores summarise how convincingly a given cluster expresses the gene set. Similarly, scCATCH [64] annotates clusters via marker gene enrichment based on a manually curated tissue-specific cell taxonomy reference CellMatch derived from a large number of tissues. Differentially expressed cluster-specific genes are identified and matched to the CellMatch database using an evidence-based scoring strategy to prioritise genes with strong support in the literature. These two methods provide highly interpretable cell identity predictions. However, both methods also share the same limitations where clustering is sensitive to parameter choice and the outcome is dependent on the quality and scope of reference marker gene resources, which limits their capacity to identify rare or previously unannotated cell types.

#### Cell-level marker and semi-supervised probabilistic models

Methods in this group take advantage of probabilistic approaches, which offers greater flexibility in cluster assignment than clustering methods (like scType or scCATCH). SCINA [67] is a method that uses an expectation–maximisation algorithm to identify the partition of cells whose expression patterns best match pre-defined marker gene sets. The rationale behind this approach is that cells genuinely belonging to a given cell type should separate cleanly from those that do not. This bimodal assumption may hold for cells in well-defined states, but transitional or undiscovered, rare cells are less likely to separate cleanly from others. Similarly, CellAssign [68] utilises a Bayesian hierarchical model that estimates the posterior probability of cells belonging to cell types defined by marker gene sets. Its Bayesian formulation accommodates noisy or overlapping markers, but the entire model still depends on the quality of the existing marker genes. CelliD [69] adopts a different strategy by projecting both genes and cells into a shared low-dimensional space. This enables the extraction of per-cell gene signatures, which are matched to known marker genes. Importantly, this enhanced granularity can be useful to improve annotation of cells in rare or transitional states.

#### Atlas-driven similarity classifiers

Tools in this group directly map query cells onto annotated reference atlases using transcriptomic similarity metrics. SingleR [70] compares each query cell to reference transcriptomes with cell type labels using correlation-based similarity. This method assumes that a cell's expression is most similar to the reference cell type it truly represents. While this simplicity in rationale enhances interpretability, a correlation-based approach does not distinguish different functional groups of genes. Similarly, Scmap [71] projects cells onto reference datasets through a nearest-neighbour approach. Scmap also allows mapping cells between different experiments by addressing technical variations. On the other hand, CHETAH [72] introduces a hierarchical cell type classification tree constructed from the reference datasets and performs cell-type annotation in a top-down manner. This method enables labelling of cells with a broad cell lineage when evidence for a more specific subtype is insufficient. While this adjustment still cannot label unknown cell types, it improves prediction robustness by annotating only cells with sufficient supporting evidence. Again, the approach is constrained by the correctness of the reference hierarchy, as misannotations in the tree can propagate.

#### Cell anchor-based label transfer

This group incorporates underlying cell representations to effectively align query cells with annotated references. For instance, Seurat v3 label transfer algorithm [3] learns cell anchors, which are pairs of mutual nearest neighbours across reference and query datasets within a joint integration space. Using these anchors, cell type labels are transferred from the reference to the query for cell-type annotation. The model assumption is that biologically similar cell states should share intrinsic transcriptomic structure across datasets, and these structures can be recovered through the anchor-based integration. This method is especially powerful for harmonising datasets from different batches and platforms. Unfortunately, its efficacy can be compromised when reference and query datasets are highly divergent or when batch effects overwhelm biological signal.

#### Conclusion

Marker or reference-based methods offer simplicity and high interpretability, making them appealing for routine cell-type annotation and quick exploratory analyses (Figure 2B). However, this narrow focus generally limits their applications primarily to direct cell-type annotation, although CelliD and Seurat can additionally support analyses of cell fate dynamics and data integration, respectively. In terms of usability, methods in this group generally provide strong user accessibility with detailed step-by-step tutorials, although their implementation typically requires basic R programming skills. Methods in this group generally require RNA-seq as the input, while some methods can take user-defined marker gene sets or reference data for the implementation (Figure 2C). As methods that directly rely on reference data, however, their performance is fundamentally limited by the completeness and accuracy of the reference atlases they depend on. Incomplete or biased references can directly lead to misclassification, especially for rare, novel, or transitional cell states. Furthermore, batch effects between query and reference datasets can distort similarity metrics, leading to dubious matching. These methods also assume that all biologically relevant cell types are present in the reference, an assumption that often fails in disease, developmental, or sparsely sampled contexts. As a result, they may produce overconfident labels in situations where ‘unknown’ would be more appropriate. Despite these limitations, their intuitive nature and transparent decision logic may appeal to many users.

### Supervised/semi-supervised machine learning methods

**Figure 3:**
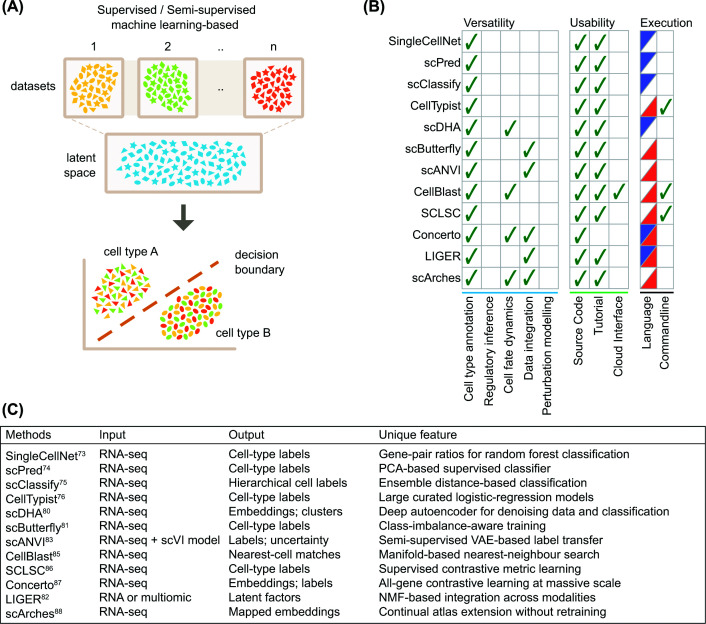
Supervised or semi-supervised machine learning methods classify cell identity in learned latent spaces. (**A**) This group identifies and utilises meaningful patterns in the data using ML-based approaches. These learned patterns are projected onto a shared latent space to perform cell-type inference analysis. (**B**) Comparison of methods for versatility, usability, and executability. Ticks indicate that a given method supports the feature. Language column denotes the programming language where the method is implemented, R (blue) or Python (red). (**C**) A summary table for input, output, and unique features of each method.

This group covers ML-based methods that do not use transformer architectures. Depending on how decision boundaries are learned for cell type prediction, methods in this group can be described as either discriminative (i.e. each cell type exhibits a distinct transcriptomic pattern) or probabilistic (i.e. cells can be seen as ones lying in continuous representations that reflect biological variation). Regardless of this classification, methods in this group achieve cell identity prediction via projecting cells onto a latent space that effectively segregates cells of different biology (Figure 3A).

#### Traditional machine learning classifiers

This sub-group utilises classical ML strategies that build cell type classifiers based on gene-cell-type relationships learned from reference transcriptomes. By projecting query cells onto these learned structures, these methods assess their biological relatedness to reference cell types. SingleCellNet [73] uses random forest classifiers trained on gene-pair features. Each gene-pair feature encodes a binary outcome: whether gene A is expressed more highly than gene B. A major benefit of the binary representation is its resistance against batch effects and cross-platform variability. This enhanced robustness supports the method’s biological rationale, where gene-pair relationships should stay stable within the same cell types, regardless of data source. SingleCellNet also flags intermediate states or hybrid cell types by providing scores for all available reference cell types, although final biological interpretation of these metrics is still left to users. Similarly, scPred [74] is well aligned with SingleCellNet in terms of its biological rationale and goal. scPred uses support vector machines built on reduced dimensions of single-cell transcriptomes and uses the most informative principal components for each cell type while regressing out technical variance in data. Its reliance on linear assumptions from the principal component analysis is a noted limitation of scPred. Lastly, scClassify [75] uses an ensemble approach with cell type hierarchy built from multiple reference data resources. Its integration of multiple discriminative models helps to improve prediction accuracy and reduce the number of unassigned cells through a post-hoc clustering procedure.

Some ML classifier methods leverage simplicity from linear learning structures for their scalability. For instance, CellTypist [76] employs logistic regression to dissect cellular heterogeneity of immune cells. Using large-scale, manually curated multi-tissue atlases as the reference for the model training, this method allows finely resolved immune cell-type annotation. The effectiveness of this method, despite its simplicity, reinforces a widely accepted view that stable biological information can be extracted independently of model complexity, provided the training data are rich and biologically relevant [77–79].

#### Deep learning-based models

Cell-type annotation can be significantly affected by non-biological and technical variations between datasets, stemming from differences in sequencing platforms, reagent kits, library preparation protocols, and independent experimental batches [78]. This sub-group primarily aims to address these unwanted factors when integrating multiple datasets, rather than solely aiming for cell identity prediction. These data harmonisation ultimately facilitates an optimal label transferring between query and reference data by reconstructing informative representations of cells while filtering out noise and technical artefacts. Methods in this group are based on the following biological rationale: an optimal data harmonisation between reference and query datasets would enable biologically meaningful cell-type annotation through label transferring, provided references are well-established and of high quality.

scDHA [80] denoises complex single-cell datasets and extracts cell-level representative information in low-dimensional embeddings. This method works optimally for large and heterogeneous datasets, but its learned embedding is intrinsically sensitive to the training set composition, and the model may propagate confounded structure (e.g., batch effects) if these dominate the training data. Furthermore, the classifier can struggle with out-of-distribution states, since the embedding space is optimised only to distinguish known classes. Similarly, scButterfly [81] improves cell-type annotation primarily by harmonising data from multimodal single-cell data. It achieves this by aligning latent spaces of different modalities (i.e. expression and chromatin structure) through training masked variational autoencoders (VAEs) to capture shared semantics (i.e. biological meaning of cells and genes) between them. By doing so, it also removes technical variations specific to each data modality. This method can be particularly useful for improving cell type prediction in incomplete datasets, as it projects well-annotated reference datasets onto them. However, it is equally important to note that its prediction occurs indirectly through a modality-translation step. As a result, errors introduced during this translation can propagate and reduce prediction accuracy, potentially making these approaches less effective than models trained directly for cell type prediction. Similarly, matrix factorisation methods such as LIGER [82] also offer a related strategy for improving cell-type annotation through better data alignment rather than direct cell type classification, albeit they rely on a linear model.

scANVI [83] addresses modality-specific bias in the reference data by building on an unsupervised data integration pipeline scVI [84]. In essence, scVI learns joint probabilistic distributions of cell transcriptomes across multiple datasets to create a shared low-dimensional latent space (i.e. a compressed cell representation), and subsequently scANVI projects existing cell type labels onto this latent space to further improve the data integration. Notably, this framework yields corrected expression values suitable for a wide range of downstream analyses, including cell clustering, differential expression, and imputation, which are all closely interleaved with cell identity analysis.

In addition, methods that utilise inter-cellular transcriptomic similarity as the basis for the cell-type annotations also fall into this group. CellBlast [85] uses cell-to-cell similarity to annotate groups sharing similar gene expression profiles derived from single-cell reference transcriptome data. By quantifying similarity between a given cell pair using a custom distance metric (i.e. normalised projection distance) specially designed for cell embeddings, it matches new cells to known cell types from the references. In doing so, the method classifies cell populations without clear matching as novel cell populations. SCLSC [86] uses supervised contrastive learning to pull cells towards their cell-type representations on the assumption that cells of the same cell type have maximal internal consistency. The learned representation of cells is then annotated with *k*-nearest neighbors for cell type prediction using the reference data. Lastly, Concerto [87] is a deep learning method that creates a unified representation of cells by learning discriminative embeddings between referenced cell types, which enables it to identify rare or disease-specific cell populations in complex samples, even those not in the referenced cell types.

Although powerful, all these deep learning-based approaches do come with significant computational burden for model training. Some methods have successfully addressed this limitation. For example, scArches [88] reuses pre-trained deep learning models but optimises only a small number of parameters necessary to align new data. This approach not only substantially reduces computational demand but also helps preserve reference integrity through sharing only required model parameters to enhance the model’s robustness. Furthermore, the model’s design inherently flags data points that do not match the reference well, allowing researchers to potentially identify novel cell types or disease states unique to their specific query dataset. This strategic application of transfer learning facilitates independent data analysis using a common reference point and accelerates iterative atlas building across the community.

#### Conclusion

Methods in this category address the non-linear complexity and technical noise inherent in single-cell data, excelling in harmonising diverse datasets by capturing intricate biological relationships. Their probabilistic representation using shared latent space makes them appropriate for large-scale data integration and robust cell-type label transfer. Our comparative analysis shows that methods with data integration generally exhibit greater versatility (Figure 3B). This suggests that the ability to effectively integrate data resources is an important determinant of a method's utility, as exemplified by Concerto and scArches. By contrast, traditional ML classifiers offer simplicity, computational efficiency, and better interpretability of biological features behind their predictions (e.g., specific gene pairs). However, these simpler models often rely on linear assumptions and fixed feature sets, which limits their robustness against major batch effects and cross-platform variability. Conversely, deep learning-based ML models, while more robust to these variations, introduce significant computational overhead, and their ‘black box’ feature renders them less interpretable. Despite the ability of ML models to capture non-linear patterns and generalise better than direct mapping approaches (e.g., marker- or reference-based methods), this group remains limited by the quality and completeness of the reference data. A novel cell state absent from the reference may be misclassified, regardless of the model’s complexity. Moreover, the reduced interpretability of these models can be a drawback in such scenarios, as the biological basis of misclassification is often far more difficult to trace compared with direct mapping methods (i.e. group 2). Despite limitations inherent to ML structure, input data requirements are relatively straightforward; all methods covered in this group take RNA-seq (Figure 3C), while the output can be various forms including cell-type labels and cell embeddings, which can be further explored for downstream analysis.

### Transformer-based methods

**Figure 4:**
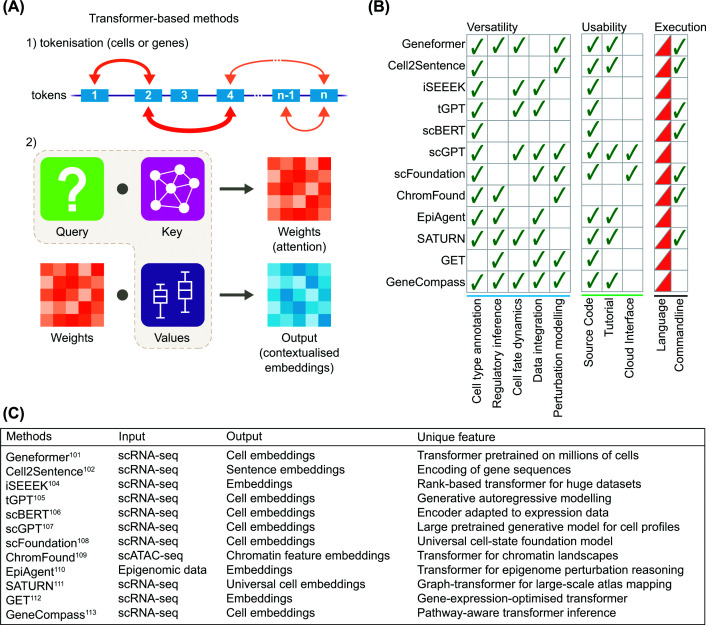
Transformer-based foundation models learn context-aware representations of cell states. (**A**) This group leverages the power of transformers for context-aware cell type inference. In the core of this approach, the attention mechanism (step 1) plays a critical role in learning contextual information between tokens (i.e. units of genes or cells). Subsequently, the transformer quantifies each token's relevance with all others by multiplying Query (i.e. what each token is looking for?) and Key (i.e. what do other tokens have things to offer?) matrices, yielding a relevance score in the attention weight matrix (step 2). By multiplying with the value matrix (i.e. actual information content), the transformer produces contextualised embeddings, which are then used for further model tuning. (**B**) Comparison of methods for versatility, usability, and executability. Ticks indicate that a given method supports the feature. Language column denotes the programming language where the method is implemented, R (blue) or Python (red). (**C**) A summary table for input, output, and unique features of each method.

In this sub-category, we examine the application of transformer-based architectures to annotate cell types. The impact of transformers in biology is best exemplified by AlphaFold [89], the Nobel-winning model for protein structure prediction. Although protein and molecular sequence models were not designed for inferring cell identities, the broader class of foundation models provides a feasible framework. These models operate on tokens, a fundamental numerical unit that represents cells by their gene expression or other molecular data (Figure 4A). While the goal of classification through cell similarity is shared between transformers and other supervised models, they have self-attention mechanisms [90,91] that reap multiple benefits where traditional models typically fall short.

One example is the unique ability to consider all cell tokens simultaneously, where traditional model architectures like recurrent or convolutional neural networks are limited to processing cells one after another, often losing crucial contextual information from cells that were processed earlier [91]. Transformers, therefore, overcome significant computational bottlenecks in information gain, revealing complex dependencies and generalising well across diverse contexts. In a cellular context, transformers generate an identity signature for each cell by precisely quantifying its unique gene profile relationship with all cells in the assayed population. The mechanistic details of transformers are well-explained elsewhere [92,93] and therefore excluded from the scope of the present review.

The first definition of foundation models was established in other domains, such as natural language processing and computer vision [94,95]. They are trained on large, heterogeneous datasets and demonstrate broad adaptability across downstream applications. Similarly, while transformer models in biology have been often constrained to a single modality, such as single-cell transcriptomics and chromatin accessibility, these datasets come in large scale and diversity, which provide theoretically sufficient heterogeneity for approximating a foundation for cellular biology [96]. This heterogeneity comes from different biological conditions captured in the data, including conditional activation of gene networks [97] and functional programs under different tissue contexts, hormonal signals, and drug perturbations [98,99]. Consequently, multi-modal training may not be strictly required to capture the breadth of cell identity. Recent large-scale single-cell transcriptomic datasets, comprising tens of millions of cells across tissues and perturbations, have enabled models to acquire broadly generalisable representations. These models can be applied directly to diverse tasks, including cell-type annotation, or further fine-tuned to achieve improved performance.

#### Three computational strategies for cell-type annotation

To enable cell-type annotation, foundation models apply three distinct computational strategies, each one processes reference transcriptomic data differently to identify cellular identities [100]. The first approach converts gene expression levels into ranked lists (i.e. ranking-based approach), recognising that cell types are often distinguished by their relative gene expression patterns rather than absolute values. The second strategy transforms continuous gene expression values into discrete categories (i.e. categorisation-based approach), converting cell-type annotation into a classification problem more familiar to traditional ML approaches. This binning strategy recognises that for cell-type annotation, the exact expression level of a gene has less biological impact than the broad expression quantile it occupies (e.g., ‘high’, ‘medium’, ‘low’). The third approach preserves the full resolution of gene expression data by directly modelling continuous expression values (i.e. continuous value-based approach). For cell-type annotation, this strategy maintains the nuanced expression differences that might distinguish closely related cell subtypes or developmental states.

Different computational approaches to cell-type annotation provide distinct advantages. Ranking-based models excel when the objective is to assess relative gene expression hierarchies. For cell-type annotation, this approach excels at capturing the relative importance of marker genes, making them well-suited for identifying cell types when the model prioritises the correct marker genes. For example, these models would recognise that in T cells, CD3 genes consistently rank higher than B cell markers like CD19, regardless of absolute expression values. Geneformer [101], built on 30 million transcriptomes, and Cell2Sentence [102] represent outstanding examples of this category. It improves on the ranking approach by focusing on predicting genes within different cellular contexts to learn gene ranking signatures corresponding to distinct cell types. This makes it particularly powerful for cell-type annotation across experimental conditions where absolute expression values might be more variable due to confounding factors. In its most recent update [103], Geneformer is currently being retrained on over 100 million transcriptomes. Other transformers include iSEEEK [104] and tGPT [105]. Although suitable for cell-type annotation, users may prefer foundational models for their generalisability, which can reflect predictions of more robust biological relationships.

Categorisation-based models demonstrate robustness to technical variation and prove effective in classifying major cell types, especially when discrete marker expression states are most informative. This strategy transforms continuous gene expression values into discrete categories, converting cell-type annotation into a classification problem similar to traditional ML approaches. This binning strategy recognises that in cell-type annotation, the exact expression level of a gene may be less important than the broad expression category it belongs to (e.g., Best and Lowest Quantile), while requiring fewer computational demands. scBERT [106] exemplifies this approach by binning gene expression values into discrete quantile buckets and treating cell-type annotation as predicting the correct expression bucket for each gene. scGPT [107] enhances this categorisation approach with attention mechanisms for autoregressive prediction, learning cell and gene representations simultaneously. It demonstrates strength for discovering novel cell subtypes, as this hybrid approach can identify cells with unexpected combinations of gene expression categories.

The third approach preserves the full resolution of gene expression data by directly modeling continuous expression values. For example, scFoundation [108] pretrains on 50 million human cells to directly project raw gene expression values using a masked autoencoder approach. For cell-type annotation, this strategy maintains the nuanced expression differences that might distinguish closely related cell subtypes or developmental states.

#### Multi-modal approaches

While expression-based strategies dominate current annotation models, emerging work points toward integrating regulatory and sequence-level signals from other biological modalities. While multimodal training may not be strictly necessary to capture most cell identities, epigenetic states are particularly important, as they reflect core aspects of cell identity as shown by the ChromFound [109] and EpiAgent [110] (i.e. single modal foundational models built on chromatin accessibility). Incorporating multiple modalities provides a more comprehensive view of cellular biology. SATURN [111] represents an early example that takes both scRNA-seq count data and protein sequences of species as input. Its central focus is to generate universal cell embeddings based on latent gene programs. It involves mapping heterogeneous datasets into a macrogene space using an autoencoder, a component of the transformer architecture. GET (general expression transformer) [112] is trained on both chromatin accessibility across 213 cell types coupled with transcriptomes of 153 cell types. Although not designed as a cell annotation model, GET provides a complementary pathway that could ultimately refine annotation in cases where scRNA-seq data alone proves insufficient. Users may map GET’s embeddings to assist cell type classification. These models illustrate a future trajectory where annotation models incorporate both expression profiles and the regulatory grammar encoded in DNA, mixing modality-specific and multimodal foundations.

GeneCompass [113] bridges all three approaches described above. In addition to ranking, it encodes absolute values and continuous expression simultaneously, layering it with a priori regulatory knowledge such as GRNs and promoter sequences. This extends the interpretability and robustness of rank-based models to cross-species annotation. On both human and mouse datasets, it consistently outperformed Geneformer [101] in F1 and accuracy, demonstrating robust biological understanding and that rank-informed representations generalise across species.

#### Conclusion

Contextual information between genes and cell states is critical to improve model generalisability beyond the biological scope covered in the reference data. In this regard, the transformer-based methods significantly benefit by leveraging the attention mechanism. Their enhanced capacity to contextualise information gives them broad utility beyond simple cell-type annotation, as reflected in our comparative analysis showing that most of these models can perform multiple related tasks (Figure 4B). While many approaches rely on single-cell transcriptomics, models built on other data modalities such as EpiAgent [110] and ChromFound [109] offer complementary regulatory insights.

Due to the extensive ML functionality available in Python packages such as TensorFlow [114], PyTorch [115], and HuggingFace [116], methods in this group are primarily implemented in Python, although many also provide command-line interfaces with adjustable parameters for easier execution. Furthermore, although these methods in this group share the transformer architecture, their motivations differ, resulting in variations in outputs and intended applications (Figure 4C). Therefore, the method choice ultimately depends on the analytical goals and the specific biological context. For example, robust classification of major cell populations may favour categorisation or marker-based methods due to clarity from the data discretisation, while rare or transitional cell types may be better resolved by ranking or projection transformers that consider continuous values. Surprisingly, for other applications such as predicting perturbation responses, a recent study demonstrates that foundation models do not consistently outperform linear models [117]. In the double perturbation prediction tasks, multiple transformer-based models, including scGPT [107], scFoundation [108], scBERT [106], and Geneformer [101], resulted in substantially higher prediction errors. This finding indicates that the practical advantages of foundation models do not necessarily translate into better classification performance. Also, these models inherit limitations from their complex model architecture: high computational demand for extensive training of a large number of parameters and limited interpretability. This observation, however, does not imply the impracticability of tuning the large number of parameters, as larger LLM models with billions of parameters still demonstrate superior cell-type annotation performance [118,119]. The real question is whether the added model complexity is justified by the performance gained: 1. how much additional computational cost is acceptable to maximise the performance? and 2. where the optimal balance between these two factors lies?

Despite these limitations, transformer-based frameworks are quickly becoming central to cell identity modelling. Their scalability, ability to integrate multimodal inputs, and capacity to generalise across datasets position them as foundation models for the next generation of cell identity analysis. As training resources continue to expand and model architectures are refined for better efficiency, transformer-based methods are expected to deliver increasingly stable, generalisable representations of cell states. This shift will likely accelerate the move toward unified models capable of handling multiple related analyses, including data integration, cell-type annotations, perturbation prediction, and regulatory inference within a single computational backbone.

### Choosing appropriate methods for analysis

Choosing suitable methods ultimately depends on the analytical goals, input data type, and available computational resources. Among these, the input data is often a key determinant of biological insights that can be derived from downstream analysis. For this reason, here we outline three common analysis scenarios to guide method selection based on input data (Table 3). These include 1. scRNA-seq with an available reference atlas, where annotation is guided by existing knowledge; 2. scRNA-seq without a reference, which focuses on discovery of novel cell states; and 3. multi-omics datasets, where the integration of complementary data modalities allows deeper biological insight. Each scenario is illustrated with representative research questions to demonstrate how multiple methods can be applied sequentially to answer them. For instance, in scenario 1, cell types may first be annotated using Seurat or SingleR, followed by regulatory inference using SCENIC or TRIAGE and then *in silico* validation by CellNet, showing how complementary tools can be applied consecutively within a single analysis workflow.

**Table 3:** Example pipeline recommendations as guidance for method selection

**Scenario 1: scRNA-seq data with an available reference atlas** Description Single-cell RNA-seq data from well-characterised tissues, where a suitable reference atlas or curated marker gene set is available for annotation. Example research question What cell types are present in a well-characterised tissue? What regulatory programs define their identity? Recommended pipeline 1. Prepare the data • Pre-processing and quality control • (Optional) Clustering of cells 2. Assign cell identities using reference • Map cells to a reference atlas or annotated dataset • Seurat, SingleR, scmap, CHETAH • (Optional) Refine cell type assignments • scType, scCATCH, SCINA, CellAssign 3. Downstream analysis using reference-based annotation • Identify key drivers • SCENIC, TRIAGE • Validate cell identity • CellNet • Trajectory modelling (e.g. cell differentiation) • CellOracle Benefits • High interpretability and reproducibility of cell type assignments • Computationally efficient Limitations • Limited capacity to identify novel cell populations • Strong dependence on the quality of the reference atlas • Bias towards pre-defined cell identities
**Scenario 2: scRNA-seq without a reference** Description Single-cell RNA-seq data from poorly characterised systems, such as developmental or disease contexts, where no reliable reference annotation is available. Example research questions What novel or previously uncharacterised cell populations exist in this system? What are their functional properties? Recommended pipeline 1. Prepare the data • Pre-processing and quality control • (Optional) Clustering of cells 2. Choose the method based on the analysis purpose • Direct cell type assignment • SingleCellNet, scPred, scClassify, CellTypist, SCLSC • Cell clustering/annotation based on cell embeddings • scANVI, scArches, scDHA, CellBalst 3. Downstream analysis for interpretation of novel states • Identify rare/ransitional cell populations • Geneformer, scGPT • Interpret newly identified cluster functionally • TRIAGE, SCENIC • Compare across datasets • scANVI, scArches Benefits • Discovery of novel, rare or transitional cell states • Flexible across diverse datasets and conditions Limitations • Reduced biological interpretability • Greater sensitivity to model assumptions during training • Requires additional validation for novel cell state
**Scenario 3: Multi-omics data (e.g. RNA-seq + ATAC-seq)** Description Multi-modal datasets combining transcriptomic and epigenomic measurements (e.g. scRNA-seq and scATAC-seq). Example research question How do gene expression and chromatin accessibility jointly define cell identity and regulatory mechanisms across conditions? Recommended pipeline 1. Prepare the data • Data pre-processing for each modality 2. Harmonisation and data integration • LIGER, scArches, scButterfly, Concerto, SATURN, scGPT, Seurat, scANVI 3. Cell identity analysis • Cell-type annotation on integrated data using available reference • Seurat • Regulatory inference • SCENIC+, CellOracle
• Cross-modal analysis using cell embedding • GeneCompass Benefits • Enhanced biological resolution through multi-layers of information • Cross-dataset and cross-condition comparisons Limitations • Increased computational and analytical complexity • Sensitivity to batch effect and modality imbalance

**Figure 5:**
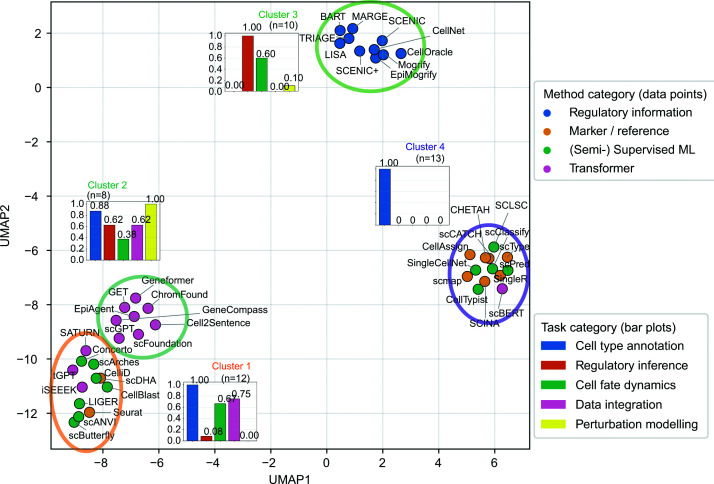
Functional clustering of 43 representative computational methods reveals specialised and versatile strategies for cell identity analysis. 43 methods from all four categories are projected on UMAP spaces based on Jaccard distance calculated from a binary matrix encoding their capacity to perform five key tasks (cell-type annotation, regulatory inference, cell fate dynamics, data integration, and perturbation modelling). This representation reveals four distinct clusters of methods with similar functional profiles. Each point represents a method coloured by its method category. For each cluster, insets show the proportion of methods supporting each task, summarising the functional composition of the clusters.

We also provide a systemic comparison of all 43 methods across four categories by visualising their functional similarity based on their capacity to support the five key tasks (Figure 5). The analysis reveals distinct clusters of methods with shared functional profiles. For instance, transformer-based methods are clustered as the most versatile group (Cluster 2), demonstrating broad capacity across all five key tasks. In contrast, cell-type annotation is supported by methods from nearly all categories, except for regulatory information-based approaches (Cluster 3), which are more specialised for regulatory inference and cell fate dynamics. A table of a complete list is also shown in Supplementary Table S2.

### Perspectives on the future development

Advancements in computational methodologies and ML have substantially increased the sophistication of cell identity models. Improved optimisation strategies, deep-learning frameworks, and the emergence of transformer-based architectures have enabled models to capture subtle patterns and complex relationships between cell states that were previously inaccessible. These developments have progressed in parallel with rapid improvements in sequencing technologies, including higher throughput, greater sensitivity, and multimodal profiling, all of which provide richer inputs for model training. However, despite these technological gains, current computational models of cell identity remain fundamentally constrained by the quality and biological diversity of reference datasets they are trained on.

In this regard, the future of computational cell identity modelling will depend less on the model’s architectural development and more on the quality of the genomic data that underpin these models. Biased or noisy datasets can propagate systematic errors and inflate prediction confidence even in incorrect predictions. As the field becomes increasingly populated with transformer-based LLMs, which offer limited transparency into how data are processed and how decisions are made within their complex structures, rigorous quality control of datasets in the first place will become increasingly important.

To address this, several community-driven initiatives have sought to improve data quality, standardisation, and accessibility. Foundational efforts including the Human Cell Atlas [4] and HuBMAP [42] established quality guidelines for metadata annotations and experimental design, while more recent studies such as CELLxGENE Discovery and the Single Cell Expression Atlas [120] and the Single Cell Expression Atlas [121,122] emphasise scalable data curation and harmonised annotation across datasets. Together with the adoption of FAIR data principles [123], these initiatives reflect a growing community recognition that high-quality, well-annotated datasets are essential for reliable model development. On this point, curated resources such as the Stemformatics repository [124] provide a concrete example of such community efforts. When applying stringent quality control criteria, they found only about 70% of processed datasets met their acceptable quality threshold. This finding highlights not only the importance of a collective effort within the research community to introduce quality standards, but also a substantial gap between the data that is available and the data needed for effective model training.

From a practical perspective, improving data quality will require closer alignment between experimental design and downstream computational use. This includes adopting standardised protocols, providing comprehensive and structured metadata, ensuring adequate biological replication, and making both raw and processed data openly accessible. Incentivising high-quality data generation may also benefit from stronger requirements from journals and funding bodies for data deposition and annotation. Together, these efforts will ensure that advances in computational modelling are aligned with the quality and reliability of the biological data that underpin them.

From the computational perspective, methods need to address current limitations in model interpretability (i.e. how does the model predict?), causality (i.e. what is causing this observation?), and explainability (i.e. why did the model make this specific prediction?) [125,126]. Deep learning and transformer methods often excel at association-based prediction, but they struggle to reveal the causal relationships that drive cell identity. To address these limitations, causal transformers [127,128], sparse autoencoders [129,130], and structure-disentangled latent models [131] can offer new ways to interpret causal links between genetic regulators and cell identity. As computational and experimental standards continue to evolve, the shift toward models that explain cell states holistically, rather than serving as simple cell-state classifiers, is expected to accelerate, but with a growing emphasis on molecular logic and the causal determinants of cell identity.

## Supplementary Material

Supplementary Tables S1-S2

## Open Access

Open access for this article was enabled by the participation of the University of Queensland in an all-inclusive Read & Publish agreement with Portland Press and the Biochemical Society under an agreement with CAUL.

## Abbreviations

ATAC: Assay for Transposase-Accessible Chromatin
GET: General Expression Transformer
GRN: Gene Regulatory Network
ML: Machine Learning
PRC2: Polycomb Repressive Complex 2
RP: Regulatory Potential
RTS: Repressive Tendency Score
TF: Transcription Factor

## References

[1] Aibar S., González-Blas C.B., Moerman T., Huynh-Thu V.A., Imrichova H., Hulselmans G. et al. (2017) SCENIC: single-cell regulatory network inference and clustering. Nat. Methods 14, 1083–1086 10.1038/nmeth.4463 28991892 PMC5937676

[2] Kamimoto K., Stringa B., Hoffmann C.M., Jindal K., Solnica-Krezel L. and Morris S.A. (2023) Dissecting cell identity via network inference and *in silico* gene perturbation. Nature 614, 742–751 10.1038/s41586-022-05688-9 36755098 PMC9946838

[3] Stuart T., Butler A., Hoffman P., Hafemeister C., Papalexi E., Mauck W.M. et al. (2019) Comprehensive integration of single-cell data. Cell 177, 1888–1902 10.1016/j.cell.2019.05.031 31178118 PMC6687398

[4] Regev A., Teichmann S.A., Lander E.S., Amt I., Benoist C., Birney E. et al. (2017) The Human Cell Atlas. eLife 6, e27041 10.7554/eLife.27041 29206104 PMC5762154

[5] Buenrostro J.D., Corces M.R., Lareau C.A., Wu B.J., Schep A.N., Aryee M.J. et al. (2018) Integrated single-cell analysis maps the continuous regulatory landscape of human hematopoietic differentiation. Cell 173, 1535.e16–1548.e16 10.1016/j.cell.2018.03.074 29706549 PMC5989727

[6] Pliner H.A., Packer J.S., McFaline-Figueroa J.L., Cusanovich D.A., Daza R.M., Aghamirzaie D. et al. (2018) Cicero predicts regulatory DNA interactions from single-cell chromatin accessibility data. Mol. Cell. 71, 858.e8–871.e8 10.1016/j.molcel.2018.06.044 30078726 PMC6582963

[7] Hwang B., Lee J.H. and Bang D. (2018) Single-cell RNA sequencing technologies and bioinformatics pipelines. Exp. Mol. Med. 50, 96 10.1038/s12276-018-0071-8 30089861 PMC6082860

[8] Ziegenhain C., Vieth B., Parekh S., Reinius B., Guillaumet-Adkins A., Smets M. et al. (2017) Comparative analysis of single-cell RNA sequencing methods. Mol. Cell. 65, 631.e4–643.e4 10.1016/j.molcel.2017.01.023 28212749

[9] Trapnell C. (2015) Defining cell types and states with single-cell genomics. Genome Res. 25, 1491–1498 10.1101/gr.190595.115 26430159 PMC4579334

[10] Stuart T. and Satija R. (2019) Integrative single-cell analysis. Nat. Rev. Genet. 20, 257–272 10.1038/s41576-019-0093-7 30696980

[11] Farrell J.A., Wang Y.Q., Riesenfeld S.J., Shekhar K., Regev A. and Schier A.F. (2018) Single-cell reconstruction of developmental trajectories during zebrafish embryogenesis. Science 360, 979 10.1126/science.aar3131 PMC6247916 29700225

[12] Wagner A., Regev A. and Yosef N. (2016) Revealing the vectors of cellular identity with single-cell genomics. Nat. Biotechnol. 34, 1145–1160 10.1038/nbt.3711 27824854 PMC5465644

[13] Mathys H., Davila-Velderrain J., Peng Z.Y., Gao F., Mohammadi S., Young J.Z. et al. (2019) Single-cell transcriptomic analysis of Alzheimer’s disease. Nature 570, 332–337 10.1038/s41586-019-1195-2 31042697 PMC6865822

[14] Puram S.V., Tirosh I., Parikh A.S., Patel A.P., Yizhak K., Gillespie S. et al. (2017) Single-cell transcriptomic analysis of primary and metastatic tumor ecosystems in head and neck cancer. Cell 171, 1611.e24–1624.e24 10.1016/j.cell.2017.10.044 29198524 PMC5878932

[15] Shekhar K., Lapan S.W., Whitney I.E., Tran N.M., Macosko E.Z., Kowalczyk M. et al. (2016) Comprehensive classification of retinal bipolar neurons by single-cell transcriptomics. Cell 166, 1308.e30–1323.e30 10.1016/j.cell.2016.07.054 27565351 PMC5003425

[16] Zeisel A., Muñoz-Manchado A.B., Codeluppi S., Lönnerberg P., La Manno G., Juréus A. et al. (2015) Cell types in the mouse cortex and hippocampus revealed by single-cell RNA-seq. Science 347, 1138–1142 10.1126/science.aaa1934 25700174

[17] Lotfollahi M., Wolf F.A. and Theis F.J. (2019) scGen predicts single-cell perturbation responses. Nat. Methods 16, 715–721 10.1038/s41592-019-0494-8 31363220

[18] Norman T.M., Horlbeck M.A., Replogle J.M., Ge A.Y., Xu A., Jost M. et al. (2019) Exploring genetic interaction manifolds constructed from rich single-cell phenotypes. Science 365, 786–793 10.1126/science.aax4438 31395745 PMC6746554

[19] Tyser R.C.V., Mahammadov E., Nakanoh S., Vallier L., Scialdone A. and Srinivas S. (2021) Single-cell transcriptomic characterization of a gastrulating human embryo. Nature 600, 285–289 10.1038/s41586-021-04158-y 34789876 PMC7615353

[20] Boycott K.M., Vanstone M.R., Bulman D.E. and MacKenzie A.E. (2013) Rare-disease genetics in the era of next-generation sequencing: discovery to translation. Nat. Rev. Genet. 14, 681–691 10.1038/nrg3555 23999272

[21] Aguet F., Barbeira A.N., Bonazzola R., Brown A., Castel S.E., Jo B. et al. (2020) The GTEx Consortium atlas of genetic regulatory effects across human tissues. Science 369, 1318–1330 10.1126/science.aaz1776 32913098 PMC7737656

[22] Boix C.A., James B.T., Park Y.P., Meuleman W. and Kellis M. (2021) Regulatory genomic circuitry of human disease loci by integrative epigenomics. Nature 590, 300–307 10.1038/s41586-020-03145-z 33536621 PMC7875769

[23] Kundaje A., Meuleman W., Ernst J., Bilenky M., Yen A., Heravi-Moussavi A. et al. (2015) Integrative analysis of 111 reference human epigenomes. Nature 518, 317–330 10.1038/nature14248 25693563 PMC4530010

[24] Moore J.E., Purcaro M.J., Pratt H.E., Epstein C.B., Shoresh N., Adrian J. et al. (2020) Expanded encyclopaedias of DNA elements in the human and mouse genomes. Nature 583, 699–710 10.1038/s41586-020-2493-4 32728249 PMC7410828

[25] Altshuler D.M., Durbin R.M., Abecasis G.R., Bentley D.R., Chakravarti A., Clark A.G. et al. (2015) A global reference for human genetic variation. Nature 526, 68–74 10.1038/nature15393 26432245 PMC4750478

[26] Dunham I., Kundaje A., Aldred S.F., Collins P.J., Davis C., Doyle F. et al. (2012) An integrated encyclopedia of DNA elements in the human genome. Nature 489, 57–74 10.1038/nature11247 22955616 PMC3439153

[27] Snyder M.P., Gingeras T.R., Moore J.E., Weng Z.P., Gerstein M.B., Ren B. et al. (2020) Perspectives on ENCODE. Nature 583, 693 10.1038/s41586-020-2449-8 32728248 PMC7410827

[28] Stunnenberg H.G., Hirst M. and Consortium I.H.E. (2016) The International Human Epigenome Consortium: a blueprint for scientific collaboration and discovery. Cell 167, 1145–1149 10.1016/j.cell.2016.11.007 27863232

[29] Adams D., Altucci L., Antonarakis S.E., Ballesteros J., Beck S., Bird A. et al. (2012) BLUEPRINT to decode the epigenetic signature written in blood. Nat. Biotechnol. 30, 224–226 10.1038/nbt.2153 22398613

[30] Barretina J., Caponigro G., Stransky N., Venkatesan K., Margolin A.A., Kim S. et al. (2012) The Cancer Cell Line Encyclopedia enables predictive modelling of anticancer drug sensitivity. Nature 483, 603–607 10.1038/nature11003 22460905 PMC3320027

[31] Forrest A.R.R., Kawaji H., Rehli M., Baillie J.K., de Hoon M.J.L., Haberle V. et al. (2014) A promoter-level mammalian expression atlas. Nature 507, 462–470 10.1038/nature13182 24670764 PMC4529748

[32] Pan L., Parini P., Tremmel R., Loscalzo J., Lauschke V.M., Maron B.A. et al. (2024) Single Cell Atlas: a single-cell multi-omics human cell encyclopedia. Genome Biol. 25, 87 10.1186/s13059-024-03246-2 38641842 PMC11027364

[33] Wang D.F., Liu S., Warrell J., Won H., Shi X., Navarro F.C.P. et al. (2018) Comprehensive functional genomic resource and integrative model for the human brain. Science 362, eaat8464 10.1126/science.aat8464 30545857 PMC6413328

[34] Jones R.C., Karkanias J., Krasnow M.A., Pisco A.O., Quake S.R., Salzman J. et al. (2022) The Tabula Sapiens: a multiple-organ, single-cell transcriptomic atlas of humans. Science 376, 711 10.1126/science.abl4896 PMC9812260 35549404

[35] Almanzar N., Antony J., Baghel A.S., Bakerman I., Bansal I., Barres B.A. et al. (2020) A single-cell transcriptomic atlas characterizes ageing tissues in the mouse. Nature 583, 590 10.1038/s41586-020-2496-1 32669714 PMC8240505

[36] Cao J.Y., O'Day D.R., Pliner H.A., Kingsley P.D., Deng M., Daza R.M. et al. (2020) A human cell atlas of fetal gene expression. Science 370, 808 10.1126/science.aba7721 PMC7780123 33184181

[37] Domcke S., Hill A.J., Daza R.M., Cao J.Y., O'Day D.R., Pliner H.A. et al. (2020) A human cell atlas of fetal chromatin accessibility. Science. 370, 809 10.1126/science.aba7612 PMC7785298 33184180

[38] Lu Z., Zhang Z., Xu Z., Abdulraouf A., Zhou W. and Cao J. (2026) Organism-wide cellular dynamics and epigenomic remodeling in mammalian aging. Science. 391, eadw6273 10.1126/science.adw627341747035

[39] Zhang Z.H., Schaefer C., Jiang W.R., Lu Z.Y., Lee J., Sziraki A. et al. (2025) A panoramic view of cell population dynamics in mammalian aging. Science. 387, 262 10.1126/science.adn3949 PMC11910726 39607904

[40] Gong Q.Y., Sharma M., Glass M.C., Kuan E.L., Chander A., Singh M. et al. (2025) Multi-omic profiling reveals age-related immune dynamics in healthy adults. Nature. 648, 415–423 10.1038/s41586-025-09686-5 PMC12711581 41162704

[41] Liang Q.N., Cheng X.S., Wang J., Owen L., Shakoor A., Lillvis J.L. et al. (2023) A multi-omics atlas of the human retina at single-cell resolution. Cell Genom. 3, 100364 10.1016/j.xgen.2023.100298 37388908 PMC10300490

[42] Snyder M.P., Lin S., Posgai A., Atkinson M., Regev A., Rood J. et al. (2019) The human body at cellular resolution: the NIH Human Biomolecular Atlas Program. Nature 574, 187–192 10.1038/s41586-019-1629-x 31597973 PMC6800388

[43] Buenostro J.D., Wu B.J., Litzenburger U.M., Ruff D., Gonzales M.L., Snyder M.P. et al. (2015) Single-cell chromatin accessibility reveals principles of regulatory variation. Nature 523, 486–490 10.1038/nature14590 26083756 PMC4685948

[44] Spitz F. and Furlong E.E.M. (2012) Transcription factors: from enhancer binding to developmental control. Nat. Rev. Genet. 13, 613–626 10.1038/nrg3207 22868264

[45] Whyte W.A., Orlando D.A., Hnisz D., Abraham B.J., Lin C.Y., Kagey M.H. et al. (2013) Master transcription factors and mediator establish super-enhancers at key cell identity genes. Cell 153, 307–319 10.1016/j.cell.2013.03.035 23582322 PMC3653129

[46] Shim W.J., Sinniah E., Xu J., Vitrinel B., Alexanian M., Andreoletti G. et al. (2020) Conserved epigenetic regulatory logic infers genes governing cell identity. Cell Syst. 11, 625 10.1016/j.cels.2020.11.001 33278344 PMC7781436

[47] Wang S., Zang C.Z., Xiao T.F., Fan J.Y., Mei S.L., Qin Q. et al. (2016) Modeling-regulation with a compendium of genome-wide histone H3K27ac profiles. Genome Res. 26, 1417–1429 10.1101/gr.201574.115 27466232 PMC5052056

[48] Kamaraj U.S., Chen J., Katwadi K., Ouyang J.F., Sun Y.B.Y., Lim Y.M. et al. (2020) EpiMogrify models H3K4me3 data to identify signaling molecules that improve cell fate control and maintenance. Cell Syst. 11, 509 10.1016/j.cels.2020.09.004 33038298

[49] Sun Y.L.Z., Shim W.J., Shen S., Sinniah E., Pham D., Su Z.Z. et al. (2023) Inferring cell diversity in single cell data using consortium-scale epigenetic data as a biological anchor for cell identity. Nucleic Acids Res. 51, e62 10.1093/nar/gkad307 37125641 PMC10287941

[50] Sinniah E., Mizikovsky D., Shim W.J., Yeung Chow C.S., Souilmi Y., Cheng F.F. et al. (2025) Conserved facultative heterochromatin across cell types identify regulatory sequences underpinning cell identity and disease. Nucleic Acids Res. 53, gkaf971 10.1093/nar/gkaf971 41224121

[51] Creyghton M.P., Cheng A.W., Welstead G.G., Kooistra T., Carey B.W., Steine E.J. et al. (2010) Histone H3K27ac separates active from poised enhancers and predicts developmental state. Proc. Natl. Acad. Sci. U.S.A. 107, 21931–21936 10.1073/pnas.1016071107 21106759 PMC3003124

[52] Wang Z.J., Civelek M., Miller C.L., Sheffield N.C., Guertin M.J. and Zang C.Z. (2018) BART: a transcription factor prediction tool with query gene sets or epigenomic profiles. Bioinformatics 34, 2867–2869 10.1093/bioinformatics/bty194 29608647 PMC6084568

[53] Qin Q., Fan J.Y., Zheng R.B., Wan C.X., Mei S.L., Wu Q. et al. (2020) Lisa: inferring transcriptional regulators through integrative modeling of public chromatin accessibility and ChIP-seq data. Genome Biol. 21, 32 10.1186/s13059-020-1934-6 32033573 PMC7007693

[54] Rao S.S.P., Huntley M.H., Durand N.C., Stamenova E.K., Bochkov I.D., Robinson J.T. et al. (2014) A 3D map of the human genome at kilobase resolution reveals principles of chromatin looping. Cell 159, 1665–1680 10.1016/j.cell.2014.11.021 25497547 PMC5635824

[55] Benayoun B.A., Pollina E.A., Ucar D., Mahmoudi S., Karra K., Wong E.D. et al. (2014) H3K4me3 breadth is linked to cell identity and transcriptional consistency. Cell 158, 673–688 10.1016/j.cell.2014.06.027 25083876 PMC4137894

[56] Rackham O.J.L., Firas J., Fang H., Oates M.E., Holmes M.L., Knaupp A.S. et al. (2016) A predictive computational framework for direct reprogramming between human cell types. Nat. Genet. 48, 331–335 10.1038/ng.3487 26780608

[57] Marku M. and Pancaldi V. (2023) From time-series transcriptomics to gene regulatory networks: a review on inference methods. PLoS Comput. Biol. 19, e1011254 10.1371/journal.pcbi.1011254 37561790 PMC10414591

[58] Cahan P., Li H., Morris S.A., da Rocha E.L., Daley G.Q. and Collins J.J. (2014) CellNet: network biology applied to stem cell engineering. Cell 158, 903–915 10.1016/j.cell.2014.07.020 25126793 PMC4233680

[59] Rauluseviciute I., Riudavets-Puig R., Blanc-Mathieu R., Castro-Mondragon J.A., Ferenc K., Kumar V. et al. (2023) JASPAR 2024: 20th anniversary of the open-access database of transcription factor binding profiles. Nucleic Acids Res. 52, D174–D182 10.1093/nar/gkad1059 PMC10767809 37962376

[60] Wingender E., Chen X., Hehl R., Karas H., Liebich I., Matys V. et al. (2000) TRANSFAC: an integrated system for gene expression regulation. Nucleic Acids Res. 28, 316–319 10.1093/nar/28.1.316 10592259 PMC102445

[61] González-Blas C.B., De Winter S., Hulselmans G., Hecker N., Matetovici I., Christiaens V. et al. (2023) SCENIC plus: single-cell multiomic inference of enhancers and gene regulatory networks. Nat. Methods 20, 1355 10.1038/s41592-023-01938-4 37443338 PMC10482700

[62] Zhao Q.Y., Shim W.J., Sun Y.L.Z., Sinniah E., Shen S.P., Boden M. et al. (2025) TRIAGE: an R package for regulatory gene analysis. Brief. Bioinform. 26, bbaf020 10.1093/bib/bbaf004 PMC11725390 39800877

[63] Ianevski A., Giri A.K. and Aittokallio T. (2022) Fully-automated and ultra-fast cell-type identification using specific marker combinations from single-cell transcriptomic data. Nat. Commun. 13, 671 10.1038/s41467-022-28803-w 35273156 PMC8913782

[64] Shao X., Liao J., Lu X.Y., Xue R., Ai N. and Fan X.H. (2020) scCATCH: automatic annotation on cell types of clusters from single-cell RNA sequencing data. iScience 23, 100882 10.1016/j.isci.2020.100882 32062421 PMC7031312

[65] Hu C.X., Li T.Y., Xu Y.Q., Zhang X.X., Li F., Bai J. et al. (2023) CellMarker 2.0: an updated database of manually curated cell markers in human/mouse and web tools based on scRNA-seq data. Nucleic Acids Res. 51, D870–D876 10.1093/nar/gkac947 36300619 PMC9825416

[66] Franzén O., Gan L.M. and Björkegren J.L.M. (2019) PanglaoDB: a web server for exploration of mouse and human single-cell RNA sequencing data. 2019, baz046 10.1093/database/baz046 30951143 PMC6450036

[67] Zhang Z., Luo D.N., Zhong X., Choi J.H., Ma Y.Q., Wang S. et al. (2019) SCINA: a semi-supervised subtyping algorithm of single cells and bulk samples. Genes (Basel) 10, 163 10.3390/genes10070531 31336988 PMC6678337

[68] Zhang A.W., O'Flanagan C., Chavez E.A., Lim J.L.P., Ceglia N., McPherson A. et al. (2019) Probabilistic cell-type assignment of single-cell RNA-seq for tumor microenvironment profiling. Nat. Methods 16, 1007–1015 10.1038/s41592-019-0529-1 31501550 PMC7485597

[69] Cortal A., Martignetti L., Six E. and Rausell A. (2021) Gene signature extraction and cell identity recognition at the single-cell level with Cell-ID. Nat. Biotechnol. 39, 1095 10.1038/s41587-021-00896-6 33927417

[70] Aran D., Looney A.P., Liu L.Q., Wu E., Fong V., Hsu A. et al. (2019) Reference-based analysis of lung single-cell sequencing reveals a transitional profibrotic macrophage. Nat. Immunol. 20, 163–172 10.1038/s41590-018-0276-y 30643263 PMC6340744

[71] Kiselev V.Y., Yiu A. and Hemberg M. (2018) scmap: projection of single-cell RNA -seq data across data sets. Nat. Methods 15, 359–362 10.1038/nmeth.4644 29608555

[72] de Kanter J.K., Lijnzaad P., Candelli T., Margaritis T. and Holstege F.C.P. (2019) CHETAH: a selective, hierarchical cell type identification method for single-cell RNA sequencing. Nucleic Acids Res. 47, gkz543 10.1093/nar/gkz543 31226206 PMC6895264

[73] Tan Y.Q. and Cahan P. (2019) SingleCellNet: a computational tool to classify single cell RNA-seq data across platforms and across species. Cell Syst. 9, 207.e2–213.e2 10.1016/j.cels.2019.06.004 31377170 PMC6715530

[74] Alquicira-Hernandez J., Sathe A., Ji H.P., Nguyen Q. and Powell J.E. (2019) scPred: accurate supervised method for cell-type classification from single-cell RNA-seq data. Genome Biol. 20, 264 10.1186/s13059-019-1862-5 31829268 PMC6907144

[75] Lin Y.X., Cao Y., Kim H.J., Salim A., Speed T.P., Lin D.M. et al. (2020) scClassify: sample size estimation and multiscale classification of cells using single and multiple reference. Mol. Syst. Biol. 16, e9398 10.15252/msb.20199389 PMC7306901 32567229

[76] Conde C.D., Xu C., Jarvis L.B., Rainbow D.B., Wells S.B., Gomes T. et al. (2022) Cross-tissue immune cell analysis reveals tissue-specific features in humans. Science 376, 713 10.1126/science.abl519 PMC7612735 35549406

[77] Abdelaal T., Michielsen L., Cats D., Hoogduin D., Mei H.L., Reinders M.J.T. et al. (2019) A comparison of automatic cell identification methods for single-cell RNA sequencing data. Genome Biol. 20, 31 10.1186/s13059-019-1795-z 31500660 PMC6734286

[78] Luecken M.D. and Theis F.J. (2019) Current best practices in single-cell RNA-seq analysis: a tutorial. Mol. Syst. Biol. 15, e8746 10.15252/msb.20188746 31217225 PMC6582955

[79] Wong D.R., Hill A.S. and Moccia R. (2025) Simple controls exceed best deep learning algorithms and reveal foundation model effectiveness for predicting genetic perturbations. Bioinformatics 41, btaf317 10.1093/bioinformatics/btaf317 40407144 PMC12202205

[80] Tran D., Nguyen H., Tran B., La Vecchia C., Luu H.N. and Nguyen T. (2021) Fast and precise single-cell data analysis using a hierarchical autoencoder. Nat. Commun. 12, 3179 10.1038/s41467-021-21312-2 33589635 PMC7884436

[81] Cao Y.C., Zhao X.M., Tang S.M., Jiang Q., Li S.J., Li S.Y. et al. (2024) scButterfly: a versatile single-cell cross-modality translation method via dual-aligned variational autoencoders. Nat. Commun. 15, 47418 10.1038/s41467-024-47418-x PMC10998864 38582890

[82] Welch J.D., Kozareva V., Ferreira A., Vanderburg C., Martin C. and Macosko E.Z. (2019) Single-cell multi-omic integration compares and contrasts features of brain cell identity. Cell 177, 1873 10.1016/j.cell.2019.05.006 31178122 PMC6716797

[83] Xu C.L., Lopez R., Mehlman E., Regier J., Jordan M. and Yosef N. (2021) Probabilistic harmonization and annotation of single-cell transcriptomics data with deep generative models. Mol. Syst. Biol. 17, e9620 10.15252/msb.20209620 33491336 PMC7829634

[84] Lopez R., Regier J., Cole M.B., Jordan M.I. and Yosef N. (2018) Deep generative modeling for single-cell transcriptomics. Nat. Methods 15, 1053–1058 10.1038/s41592-018-0229-2 30504886 PMC6289068

[85] Cao Z.J., Wei L., Lu S., Yang D.C. and Gao G. (2020) Searching large-scale scRNA-seq databases via unbiased cell embedding with Cell BLAST. Nat. Commun. 11, 3452 10.1038/s41467-020-17281-7 32651388 PMC7351785

[86] Heryanto Y.D., Zhang Y.Z. and Imoto S. (2024) Predicting cell types with supervised contrastive learning on cells and their types. Sci. Rep. 14 25691 10.1038/s41598-023-50185-2 38172501 PMC10764802

[87] Yang M., Yang Y.Y.X., Xie C.X., Ni M., Liu J., Yang H.M. et al. (2022) Contrastive learning enables rapid mapping to multimodal single-cell atlas of multimillion scale. Nat. Mach. Intell. 4, 696 10.1038/s42256-022-00518-z

[88] Lotfollahi M., Naghipourfar M., Luecken M.D., Khajavi M., Buettner M., Wagenstetter M. et al. (2022) Mapping single-cell data to reference atlases by transfer learning. Nat. Biotechnol. 40, 121–130 10.1038/s41587-021-01001-7 34462589 PMC8763644

[89] Jumper J., Evans R., Pritzel A., Green T., Figurnov M., Ronneberger O. et al. (2021) Highly accurate protein structure prediction with AlphaFold. Nature 596, 583–589 10.1038/s41586-021-03819-2 34265844 PMC8371605

[90] Raiaan M.A.K., Mukta M.S.H., Fatema K., Fahad N.M., Sakib S., Mim M.M.J. et al. (2024) A review on large language models: architectures, applications, taxonomies, open issues and challenges. IEEE Access 12, 26839–26874 10.1109/ACCESS.2024.3365742

[91] Vaswani A., Shazeer N., Parmar N., Uszkoreit J., Jones L., Gomez A.N. et al. (2017) Attention is all you need. Adv. Neur. In. 30 5998–6008 10.48550/arxiv.1706.03762

[92] Chen R.J., Ding T., Lu M.Y., Williamson D.F.K., Jaume G., Song A.H. et al. (2024) Towards a general-purpose foundation model for computational pathology. Nat. Med. 30, 850–862 10.1038/s41591-024-02857-3 38504018 PMC11403354

[93] Guo F., Guan R.C., Li Y.H., Liu Q., Wang X.W., Yang C. et al. (2025) Foundation models in bioinformatics. Natl. Sci. Rev. 12, nwaf028 10.1093/nsr/nwaf028 40078374 PMC11900445

[94] Radford A., Narasimhan K., Salimans T. and Sutskever I. (2018) Improving language understanding by generative pre-training

[95] Devlin J., Chang M.-W., Lee K. and Toutanova K.(eds). (2019) Bert: Pre-training of deep bidirectional transformers for language understanding. In Proceedings of the 2019 Conference of the North American Chapter of the Association for Computational Linguistics: Human Language Technologies, Vol. 1 (Long and Short Papers), 4171–4186 Association for Computational Linguistics, Minneapolis, MN, USA 10.18653/v1/N19-1423

[96] Qin S.Y., Yang Y.X., Peng L.M., Xu L.L., Deng Y.S., Wang H.Y. et al. (2025) scGeneBank: cross-species screening of functional gene sets at single-cell resolution. Nucleic Acids Res. 54, D1251–D1269 10.1093/nar/gkaf1137 PMC12807785 41251182

[97] Russell M., Aqil A., Saitou M., Gokcumen O. and Masuda N. (2023) Gene communities in co-expression networks across different tissues. PLoS Comput. Biol. 19, e1011616 10.1371/journal.pcbi.1011616 37976327 PMC10691702

[98] Subramanian A., Narayan R., Corsello S.M., Peck D.D., Natoli T.E., Lu X.D. et al. (2017) A next generation connectivity map: L1000 platform and the first 1,000,000 profiles. Cell 171, 1437.e17–1452.e17 10.1016/j.cell.2017.10.049 29195078 PMC5990023

[99] Wira C.R., Rodriguez-Garcia M. and Patel M.V. (2015) The role of sex hormones in immune protection of the female reproductive tract. Nat. Rev. Immunol. 15, 217–230 10.1038/nri3819 25743222 PMC4716657

[100] Zeng Y.S., Xie J.C., Shangguan N.Y., Wei Z.Y., Li W.B., Su Y. et al. (2025) CellFM: a large-scale foundation model pre-trained on transcriptomics of 100 million human cells. Nat. Commun. 16, 59926 10.1038/s41467-025-59926-5 PMC12092794 40393991

[101] Theodoris C.V., Xiao L., Chopra A., Chaffin M.D., Al Sayed Z.R., Hill M.C. et al. (2023) Transfer learning enables predictions in network biology. Nature 618, 353–359 10.1038/s41586-023-06139-9 37258680 PMC10949956

[102] Levine D., Rizvi S.A., Lévy S., Pallikkavaliyaveetil N., Zhang D., Chen X. et al. (2024) Cell2Sentence: teaching large language models the language of biology. Proceedings of Machine Learning Research. 235, 27299–27325 10.5555/3692070.3693159

[103] Chen H., Venkatesh M.S., Ortega J.G., Mahesh S.V., Nandi T.N., Madduri R.K. et al. (2024) Quantized multi-task learning for context-specific representations of gene network dynamics. bioRxiv. 2024.08.16.608180 10.1101/2024.08.16.608180

[104] Shen H.R., Shen X.L., Feng M.Y., Wu D., Zhang C., Yang Y.C. et al. (2022) A universal approach for integrating super large-scale single-cell transcriptomes by exploring gene rankings. Brief. Bioinform. 23, bbab573 10.1093/bib/bbab573 35048121

[105] Shen H.R., Liu J.L., Hu J.N., Shen X.L., Zhang C., Wu D. et al. (2023) Generative pretraining from large-scale transcriptomes for single-cell deciphering. iScience 26, 106536 10.1016/j.isci.2023.106536 37187700 PMC10176267

[106] Yang F., Wang W.C., Wang F., Fang Y., Tang D.Y., Huang J.Z. et al. (2022) scBERT as a large-scale pretrained deep language model for cell-type annotation of single-cell RNA-seq data. Nat. Mach. Intell. 4, 852 10.1038/s42256-022-00534-z

[107] Cui H.T., Wang C., Maan H., Pang K., Luo F.N., Duan N. et al. (2024) scGPT: toward building a foundation model for single-cell multi-omics using generative AI. Nat. Methods 21, 02201–0 10.1038/s41592-024-02201-0 38409223

[108] Hao M.S., Gong J., Zeng X., Liu C.M., Guo Y.C., Cheng X.Y. et al. (2024) Large-scale foundation model on single-cell transcriptomics. Nat. Methods 21, 02305–7 10.1038/s41592-024-02305-7 38844628

[109] Jiao Y., Liu Y., Zhang Y., Guo X., Wu Y., Jiang C. et al. (2025) ChromFound: towards a universal foundation model for single-cell chromatin accessibility data. arXiv. 250512638 10.48550/arXiv.2505.12638

[110] Chen X.Y., Li K.Y., Cui X.J., Wang Z., Jiang Q., Lin J.C. et al. (2025) EpiAgent: foundation model for single-cell epigenomics. Nat. Methods. 22, 1127–1138 10.1038/s41592-025-02822-z 40999099

[111] Rosen Y., Brbic M., Roohani Y., Swanson K., Li Z. and Leskovec J. (2024) Toward universal cell embeddings: integrating single-cell RNA-seq datasets across species with SATURN. Nat. Methods 21, 02191–z 10.1038/s41592-024-02191-z PMC11310084 38366243

[112] Fu X., Mo S.T., Buendia A., Laurent A.P., Shao A.Q., Alvarez-Torres M.D. et al. (2025) A foundation model of transcription across human cell types. Nature 637, 08391–z 10.1038/s41586-024-08391-z PMC11754112 39779852

[113] Yang X.D., Liu G.L., Feng G.H., Bu D.C., Wang P.F., Jiang J. et al. (2024) GeneCompass: deciphering universal gene regulatory mechanisms with a knowledge-informed cross-species foundation model. Cell Res. 34, 1088–1104 10.1038/s41422-024-01034-y PMC11615217 39375485

[114] Abadi M., Agarwal A., Barham P., Brevdo E., Chen Z., Citro C. et al. (2016) Tensorflow: large-scale machine learning on heterogeneous distributed systems. arXiv. 160304467 10.48550/arXiv.1603.04467

[115] Paszke A., Gross S., Massa F., Lerer A., Bradbury J., Chanan G. et al. (2019) Pytorch: An imperative style, high-performance deep learning library. Adv. Neural Inf. Process. Syst. 32 240–250 10.48550/arXiv.1912.01703

[116] Wolf T., Debut L., Sanh V., Chaumond J., Delangue C., Moi A. et al.(eds). (2020) Transformers: state-of-the-art natural language processing. In Proceedings of the 2020 Conference on Empirical Methods in Natural Language Processing: System Demonstrations,38–45 Association for Computational Linguistics Stroudsburg, Pennsylvania, USA 10.18653/v1/2020.emnlp-demos.6

[117] Ahlmann-Eltze C., Huber W. and Anders S. (2025) Deep-learning-based gene perturbation effect prediction does not yet outperform simple linear baselines. Nat. Methods 22, 1657–1661 10.1038/s41592-025-02772-6 40759747 PMC12328236

[118] Rizvi S.A., Levine D., Patel A., Zhang S., Wang E., He S. et al. (2025) Scaling large language models for next-generation single-cell analysis. bioRxiv. 648850 10.1101/2025.04.14.648850

[119] Yang C., Zhang X. and Chen J. (2025) Large language model consensus substantially improves the cell type annotation accuracy for scRNA-seq data. bioRxiv. 647852 10.1101/2025.04.10.647852 42260142

[120] Abdulla S., Aevermann B., Assis P., Badajoz S., Bell S.M., Bezzi E. et al. (2024) CZ CELLxGENE Discover: a single-cell data platform for scalable exploration, analysis and modeling of aggregated data. Nucleic Acids Res. 53, D886–D900 10.1093/nar/gkae1142 PMC11701654 39607691

[121] George N., Fexova S., Fuentes A.M., Madrigal P., Bi Y.L., Iqbal H. et al. (2023) Expression Atlas update: insights from sequencing data at both bulk and single cell level. Nucleic Acids Res. 52, D107–D114 10.1093/nar/gkad1021 PMC10767917 37992296

[122] Papatheodorou I., Moreno P., Manning J., Fuentes A.M.P., George N., Fexova S. et al. (2020) Expression Atlas update: from tissues to single cells. Nucleic Acids Res. 48, D77–D83 10.1093/nar/gkz947 31665515 PMC7145605

[123] Wilkinson M.D., Dumontier M., Aalbersberg I.J., Appleton G., Axton M., Baak A. et al. (2019) The FAIR Guiding Principles for scientific data management and stewardship. Sci. Data 3, 160018 10.1038/sdata.2016.18 26978244 PMC4792175

[124] Choi J., Pacheco C.M., Mosbergen R., Korn O., Chen T., Nagpal I. et al. (2019) Stemformatics: visualize and download curated stem cell data. Nucleic Acids Res. 47, D841–D846 10.1093/nar/gky1064 30407577 PMC6323943

[125] Chen V., Yang M.Y., Cui W.B., Kim J.S., Talwalkar A. and Ma J. (2024) Applying interpretable machine learning in computational biology-pitfalls, recommendations and opportunities for new developments. Nat. Methods 21, 1454–1461 10.1038/s41592-024-02359-7 39122941 PMC11348280

[126] Holzinger A., Langs G., Denk H., Zatloukal K. and Müller H. (2019) Causability and explainability of artificial intelligence in medicine. Wires Data Min. Knowl. 9, e1312 10.1002/widm.1312 PMC7017860 32089788

[127] Li Z., Xu Y., Chowdhury D., Yip H.F., Wang C. and Zhang L. (2025) Causal transformer for learning embeddings from structured medical history records and multi-source data integration for complex disease risk prediction. Interdiscip. Sci. 17, 1–16 10.1007/s12539-025-00749-9 40963070 PMC13219203

[128] Melnychuk V., Frauen D. and Feuerriegel S.(eds). (2022) Causal transformer for estimating counterfactual outcomes. In International Conference on Machine Learning, 162 15293–15329 PMLR 10.48550/arXiv.2204.07258

[129] Cunningham H., Ewart A., Riggs L., Huben R. and Sharkey L. (2023) Sparse autoencoders find highly interpretable features in language models. arXiv. 230908600 10.48550/arXiv.2309.08600

[130] Gujral O., Bafna M., Alm E. and Berger B. (2025) Sparse autoencoders uncover biologically interpretable features in protein language model representations. Proc. Natl. Acad. Sci. U.S.A. 122, e2506316122 10.1073/pnas.2506316122 40828027 PMC12403088

[131] Esmaeili B., Wu H., Jain S., Bozkurt A., Siddharth N., Paige B. et al.(eds). (2019) Structured disentangled representations. In The 22nd International Conference on Artificial Intelligence and Statistics, 89 2525–2534 PMLR https://proceedings.mlr.press/v89/esmaeili19a.html

[132] Mei S.L., Qin Q., Wu Q., Sun H.F., Zheng R.B., Zang C.Z. et al. (2017) Cistrome Data Browser: a data portal for ChIP-Seq and chromatin accessibility data in human and mouse. Nucleic Acids Res. 45, D658–D662 10.1093/nar/gkw983 27789702 PMC5210658

[133] Yuan H.T., Yan M., Zhang G.X., Liu W., Deng C.Y., Liao G.M. et al. (2019) CancerSEA: a cancer single-cell state atlas. Nucleic Acids Res. 47, D900–D908 10.1093/nar/gky939 30329142 PMC6324047

