# Supplementary material for: Computational modelling of cell identity
Source: Biochem J. 2026 May 22;483(6):1049–71. doi: 10.1042/BCJ20250132 (PMC13199844; doi:10.1042/BCJ20250132)
Supplement: Supplementary Tables S1-S2 [file BCJ-2025-0132C_supp.pdf]

Tool	Source_code
TRIAGE	https://github.com/palpant-comp/TRIAGE_R_Package
MARGE	http://cistrome.org/MARGE/index.html
EpiMogrify	NA
LISA	https://github.com/qinqian/lisa
BART	https://github.com/zanglab/bart2
Mogrify	NA
CellNet	https://github.com/CahanLab/cellnetr
SCENIC	https://github.com/aertslab/SCENIC
SCENIC+	https://github.com/aertslab/scenicplus
CellOracle	https://github.com/morris-lab/CellOracle
scType	https://github.com/IanevskiAleksandr/sc-type
scCATCH	https://github.com/ZJUFanLab/scCATCH
SCINA	https://github.com/jcao89757/SCINA
CellAssign	https://github.com/Irrationone/cellassign
CellID	https://github.com/RausellLab/CelliD
SingleR	https://github.com/dviraran/SingleR
scmap	https://github.com/hemberg-lab/scmap
CHETAH	https://github.com/jdebru/chetah
Seurat	https://github.com/satijalab/seurat
SingleCellNe	https://github.com/pcahan1/SingleCellNet
scPred	https://github.com/powellgenomicslab/scPred
scClassify	https://github.com/SydneyBioX/scClassify
CellTypist	https://github.com/Teichlab/celltypist
scDHA	https://github.com/duct317/scDHA
scButterfly	https://github.com/BioX-NKU/scButterfly
scANVI	https://github.com/scverse/scvi-tools
CellBlast	https://github.com/gao-lab/Cell_BLAST
SCLSC	https://github.com/yaozhong/SCLSC
Concerto	https://github.com/melobio/Concerto-reproducibility
LIGER	https://github.com/welch-lab/liger
scArches	https://github.com/theislab/scarches
Geneformer	https://github.com/jkobject/geneformer
Cell2Senten	https://github.com/vandijklab/cell2sentence
iSEEEK	https://github.com/lixiangchun/iSEEEK
tGPT	https://github.com/deeplearningplus/tGPT
scBERT	https://github.com/TencentAILabHealthcare/scBERT
scGPT	https://github.com/bowang-lab/scGPT
scFoundatio	https://github.com/biomap-research/scFoundation
ChromFound	
EpiAgent	https://github.com/xy-chen16/EpiAgent
SATURN	https://github.com/snap-stanford/saturn
GET	https://github.com/GET-Foundation
GeneCompa	https://github.com/xCompass-AI/GeneCompass

Additional_information(tutorial)

https://triage-r-package.readthedocs.io/en/latest/index.html

https://mogrify.co.uk/science/epimogrify/

https://mogrify.net

http://cellnet.hms.harvard.edu

https://sctype.app

http://lce.biohpc.swmed.edu/scina

https://irrationone.github.io/cellassign/articles/introduction-to-cellassign.html

https://scmap.cog.sanger.ac.uk

https://www.asap.epfl.ch/seurat-wizard

https://powellgenomicslab.github.io/scPred/articles/introduction.html

https://sydneybiox.github.io/scClassify/index.html

https://colab.research.google.com/github/Teichlab/celltypist/blob/main/docs/notebo

http://scdha.tinnguyen-lab.com

https://scbutterfly.readthedocs.io/en/latest/

https://docs.scvi-tools.org/en/stable/user_guide/models/scanvi.html

https://cblast.readthedocs.org/

https://welch-lab.github.io/liger/articles/Integrating_multi_scRNA_data.html

https://docs.scarches.org/en/latest/

https://geneformer.readthedocs.io/en/latest/

https://vandijklab-cell2sentence.readthedocs.io/

https://github.com/bowang-lab/scGPT/blob/main/tutorials/zero-shot/Tutorial_ZeroSh

https://github.com/xy-chen16/EpiAgent/blob/main/demo/Data%20Preprocessing.ipyr

https://github.com/xCompass-AI/GeneCompass/blob/main/downstream_tasks/examp

CloudInterface

http://lisa.cistrome.org
http://bartweb.org

https://scenic.aertslab.org

ok/celltypist_tutorial.ipynb

http://cblast.gao-lab.org/

https://app.superbio.ai/apps/6548f339a9ed6f6e5560b07d
https://xtrimo.en.biomap.com/

b

les/celltype_annotation.ipynb

Comparison of methods

Method	Cell type annotation	Regulatory inference	Cell fate dynamics
TRIAGE	No	Yes	No
MARGE	No	Yes	No
EpiMogrify	No	Yes	Yes
LISA	No	Yes	No
BART	No	Yes	No
Mogrify	No	Yes	Yes
CellNet	No	Yes	Yes
SCENIC	No	Yes	Yes
SCENIC+	No	Yes	Yes
CellOracle	No	Yes	Yes
scType	Yes	No	No
scCATCH	Yes	No	No
SCINA	Yes	No	No
CellAssign	Yes	No	No
CelliD	Yes	No	Yes
SingleR	Yes	No	No
scmap	Yes	No	No
CHETAH	Yes	No	No
Seurat	Yes	No	No
SingleCellNet	Yes	No	No
scPred	Yes	No	No
scClassify	Yes	No	No
CellTypist	Yes	No	No
scDHA	Yes	No	Yes
scButterfly	Yes	No	No
scANVI	Yes	No	No
CellBlast	Yes	No	Yes
SCLSC	Yes	No	No
Concerto	Yes	No	Yes
LIGER	Yes	No	No
scArches	Yes	No	Yes
Geneformer	Yes	Yes	Yes
Cell2Sentence	Yes	No	No
iSEEEK	Yes	No	Yes
tGPT	Yes	No	Yes
scBERT	Yes	No	No
scGPT	Yes	No	Yes
scFoundation	Yes	No	No
ChromFound	Yes	Yes	No
EpiAgent	Yes	Yes	No
SATURN	Yes	Yes	Yes
GET	No	Yes	No
GeneCompass	Yes	Yes	Yes

Data integration	Perturbation modelling	Source Code	Tutorial	Cloud Interface
No	No	Yes	Yes	No
No	No	Yes	Yes	No
No	No	No	Yes	No
No	No	Yes	Yes	Yes
No	No	Yes	Yes	Yes
No	No	No	Yes	No
No	No	Yes	Yes	Yes
No	No	Yes	Yes	No
No	No	Yes	Yes	No
No	Yes	Yes	Yes	No
No	No	Yes	Yes	Yes
No	No	Yes	Yes	No
No	No	Yes	Yes	No
No	No	Yes	Yes	No
No	No	Yes	Yes	No
No	No	Yes	Yes	No
No	No	Yes	Yes	No
No	No	Yes	No	No
Yes	No	Yes	Yes	No
No	No	Yes	Yes	No
No	No	Yes	Yes	No
No	No	Yes	Yes	No
No	No	Yes	Yes	No
No	No	Yes	Yes	No
Yes	No	Yes	Yes	No
Yes	No	Yes	Yes	No
No	No	Yes	Yes	Yes
No	No	Yes	Yes	No
Yes	No	Yes	No	No
Yes	No	Yes	Yes	No
Yes	No	Yes	Yes	No
No	Yes	Yes	Yes	No
No	Yes	Yes	Yes	No
Yes	No	Yes	No	No
Yes	No	Yes	No	No
No	No	Yes	No	No
Yes	Yes	Yes	Yes	Yes
Yes	Yes	Yes	No	Yes
No	Yes	No	No	No
Yes	Yes	Yes	Yes	No
Yes	No	Yes	Yes	No
Yes	Yes	Yes	No	No
Yes	Yes	Yes	Yes	No

Language	Commandline
R and Python	Yes
Python	Yes
NA	No
Python	Yes
Python	Yes
NA	No
R	No
Python	Yes
Python	No
Python	No
R	No
R	No
R	No
R	No
R	No
R	No
R	No
R	No
R	No
R	No
R	No
R	No
Python	Yes
R	No
Python	No
Python	No
Python	Yes
Python	Yes
R and Python	No
R and Python	No
Python	No
Python	Yes
Python	Yes
Python	No
Python	Yes
Python	Yes
Python	No
Python	Yes
Python	Yes
Python	No
Python	Yes
Python	No
Python	No
